# Species accumulation in small–large vs large–small order: more species but not all species?

**DOI:** 10.1007/s00442-022-05261-1

**Published:** 2022-09-17

**Authors:** David C. Deane

**Affiliations:** 1 Research Centre for Future Landscapes, Department of Environment and Genetics, La Trobe University, Bundoora, VIC 3083 Australia; 2 Department of Renewable Resources, University of Alberta, Edmonton, AB T6G 2R3 Canada

**Keywords:** Diversity, Fragmentation, Island, Species–area relationship, SLOSS

## Abstract

Although groups of small habitat patches often support more species than large patches of equal total area, their biodiversity value remains controversial. An important line of evidence in this debate compares species accumulation curves, where patches are ordered from small–large and large–small (aka ‘SLOSS analysis’). However, this method counts species equally and is unable to distinguish patch size dependence in species’ occupancies. Moreover, because of the species–area relationship, richness differences typically only contribute to accumulation in small–large order, maximizing the probability of adding species in this direction. Using a null model to control for this, I tested 202 published datasets from archipelagos, habitat islands and fragments for patch size dependence in species accumulation and compared conclusions regarding relative species accumulation with SLOSS analysis. Relative to null model expectations, species accumulation was on average 2.7% higher in large–small than small–large order. The effect was strongest in archipelagos (5%), intermediate for fragments (1.5%) and smallest for habitat islands (1.1%). There was no difference in effect size among taxonomic groups, but each shared this same trend. Results suggest most meta-communities include species that either prefer, or depend upon, larger habitat patches. Relative to SLOSS analysis, null models found lower frequency of greater small-patch importance for species representation (e.g., for fragments: 69 vs 16% respectively) and increased frequency for large patches (fragments: 3 vs 25%). I suggest SLOSS analysis provides unreliable inference on species accumulation and the outcome largely depends on island species–area relationships, not the relative diversity value of small vs large patches.

**Supplementary Information:**

The online version contains supplementary material available at 10.1007/s00442-022-05261-1.

## Introduction

It is a common empirical finding that several small habitat patches contain more species than a single large patch of equal total area (Fahrig 2017, 2020; Quinn and Harrison 1988), yet why such a pattern so frequently arises remains largely unexplained (Deane et al. 2020; Fahrig 2017, 2020; Fahrig et al. 2021). That higher richness would be observed in groups of small patches for a given total area is counterintuitive, given the body of theoretical and empirical evidence of negative impacts of patch area for diversity (e.g., Chase et al. 2020; Fletcher et al. 2018; Haddad et al. 2015). In the context of fragmentation, one explanation is that small patches accumulate only more common generalist or matrix species (Andrén 1994; Matthews et al. 2014; McCollin 1993), limiting any conservation value (Blake and Karr 1984). One line of evidence that consistently supports greater species richness among groups of small patches is the comparison of species accumulation curves, where patches are ordered from the smallest to the largest and the reverse (often called ‘SLOSS analysis’; Fahrig 2017, 2020). However, this method does not account for any patch size dependence in species’ occupancies and cannot address the question of whether patches of all sizes provide equivalent habitat value for all species. Moreover, the comparison is inherently flawed because richness differences among patches can only contribute to species accumulation in small–large order and because scale dependence in quantifying species richness is ignored. However, a suitable null model can account for these issues, allowing a test of the role of patch size for species representation in the landscape using these same data.

The method of combining accumulation curves in reverse size order was introduced by Quinn and Harrison (1988) and, despite criticism (see Electronic Supplemental Material, Online resource 1), remains popular for qualitative comparisons (e.g., Richardson et al. 2015), to test assembly hypotheses (Liu et al. 2018; MacDonald et al. 2018a) and to infer the effect of habitat subdivision on species richness (Fahrig 2017, 2020). Although the Quinn & Harrison method (hereafter QH curves) treats all species equally, one can separately analyze rare or specialist species and/or common and generalist species and compare their patterns of accumulation with area. Both Rösch et al. (2015) and Fahrig (2020), used this approach to show small–large curves typically accumulated species more rapidly than large–small curves even among specialist species. Notably, this contrasts with Matthews et al. (2014), who found island species–area curves for specialist bird species were steeper than that of generalist species, suggesting greater area dependence. The finding also runs counter to empirical evidence that some specialist species are disadvantaged in smaller patches because of the limited core habitat available (Didham et al. 1998; Pfeifer et al. 2017). While a focus on local, rather than landscape, scale understanding of species richness likely contributes to these conflicting results (Fahrig et al. 2021), there is also good reason to question any inference from QH curves.

Indeed, since their introduction QH curves have been controversial (Online resource 1), not least because of controversy over the original statistical test (Fletcher et al. 2018; Mac Nally and Lake 1999). There are, however, also more fundamental problems in directly comparing species density (i.e., the number of species for a given area) when combining irregularly sized patches in reverse size order. The curves amount to a race over the same total area to encounter all species in the dataset. In general, as samples (here patches) are combined, new species can be encountered either because of turnover in species identity or due to differences in species richness (reviewed in Legendre 2014). Because of the species–area relationship, small–large accumulation of patches will typically mean the larger patch contains more species; but any new species accumulated due to differences in richness between the patches can only contribute to species accumulation in small–large order. As a result, the probability of encountering new species is maximized for every patch when combining them in small–large order (Online resource 2). The combination of small patches, each with maximized probability of encountering new species accounts for the rapid initial accumulation of species typical of the small–large curve (Quinn and Harrison 1988). Greater large–small accumulation is only possible when the largest patch contains a suitably high proportion of total species richness in the data, which is most likely to occur when the island species–area relationship is steep (e.g., high slope values for the power-law species–area model), or the largest patch contains a high proportion of total habitat area.

**Fig. 1:**
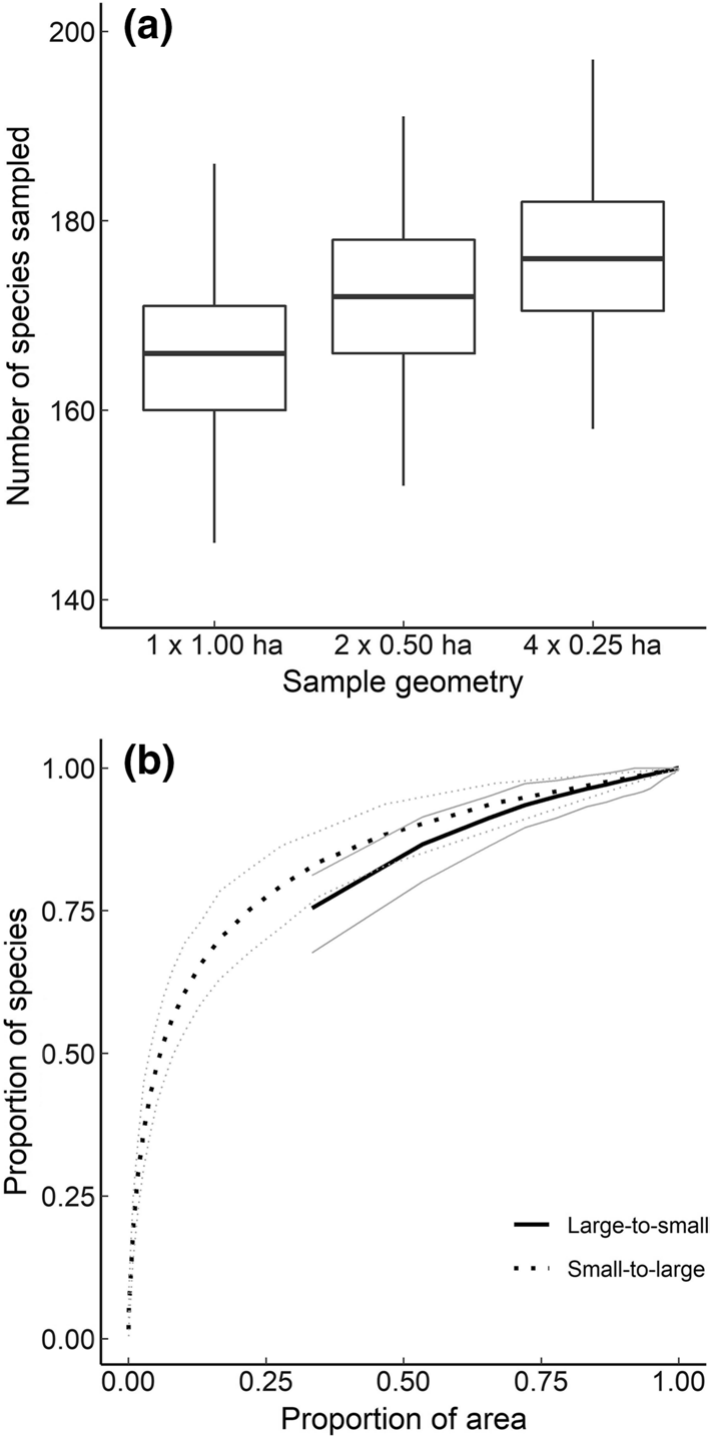
Effect of different sampling geometry on expected species richness within a continuous forest tree community. **a** Distribution of richness values for 200 randomly positioned sampling units of total 1-ha area divided into 1, 2 or 4 quadrats. **b** Variation in accumulated species richness for 20 irregularly sized quadrats combined in large-to-small (LTS) and small-to-large (STL) order. Black lines show mean number of species accumulated over 200 randomly positioned iterations, gray lines show 95% sampling intervals. Sample size distribution in (**b**) based on log-normal distribution (see Online resource 2) and a total sampled area of 2.5 ha. Data: Barro Colorado Island forest plot, 2005 census (Condit et al. 2012)

A second problem with QH curves arises from the purely geometric effects of habitat subdivision (May et al. 2019). It is well known that the scale at which an ecological phenomenon is investigated influences the pattern that is observed (Wiens 1989). If most species in an assemblage are aggregated in space, a group of small patches will typically contain more species than an equivalent area contained in a single patch, which can be shown analytically (Deane et al. 2022; Kobayashi 1985). It can also be illustrated (Fig. 1) using stem-mapped forest data such as the 50-ha Barro Colorado Island forest dynamics plot (Condit et al. 2012). Essentially, QH curves overlook the need to account for the effects of scale when seeking to understand species richness in disjoint habitats (Chase et al. 2019; Chase et al. 2018; Giladi et al. 2014). To identify any underlying ecological mechanism for the accumulation of species, including patch size dependence, it is necessary to control for any sampling effects on the species–area relationship (Chase et al. 2019; Hill et al. 1994), which can be achieved with a null model.

The simplest explanation assumes the species–area relationship arises as a passive sampling phenomenon (Connor and McCoy 1979), where the probability of observing a species in a patch jointly depends on the size of the sample (e.g., the number of individuals) and the relative abundance of the species in the landscape. While strictly relating to random placement of individuals (Arrhenius 1921; Coleman 1981), if we assume occupancy and abundance are positively correlated, this can be implemented as a null model for presence–absence data using an algorithm that retains the total number of occupancies and total richness of individual sites (i.e., constant row and column sums). If all points in the observed small–large and large–small curves fall within a randomization envelope generated using the null model, we are unable to reject a hypothesis of passive sampling. Because the algorithm removes any patch size dependence on species’ occupancies, the sampling envelope simulates situations where species accumulation was not affected by patch size. If either curve falls outside the randomization envelope, it suggests some patch size dependence in occupancy affects species accumulation (Methods).

The aims of this paper are threefold. First, to test for evidence of deviation from passive sampling when combining sites in reverse size order, implying some influence of patch size on species accumulation not revealed from QH curves. Second, to test for any systematic deviations from passive sampling for species accumulation related to broad meta-community type (archipelagos, fragments, habitat islands) or taxonomic group (plants, invertebrates, birds, non-avian vertebrates). Finally, to test the sensitivity of conclusions from null models and SLOSS analysis to other meta-community covariates, focusing on nestedness and the slope of the island species–area relationship.

## Materials and methods

### Sources of data

I compiled datasets from published studies including raw data for discrete habitat types differing in patch area, building on the database from an earlier study compiled using literature searches and citation tracking as described in Deane and He (2018). I included ‘true’ islands (hereafter archipelagos and including those of inland, continental and oceanic waters), habitat islands (e.g., lakes, wetlands, sky islands) and remnant fragments of forest, woodland or grassland. In total, I acquired 202 presence-absence datasets (see data sources in Online resource 3 and associated metadata and results in Online resource 4). I made no assumption on sampling effort per patch, other than to assume each patch provided a comparable representation of species richness within the patch for that study system. However, the outcome of SLOSS analysis is sensitive to survey effort (Deane et al. 2020; Fahrig 2020) and the methods of sampling varied widely between studies. Datasets (hereafter meta-communities) were therefore given an ordinal classification according to the level of confidence the data constituted a full census of species present in each patch, with results tested for sensitivity to these data confidence categories. The criteria were: (1, highest confidence) atlas data or field confirmed atlas data; (2) multiple survey methods or collation of multiple field visits; (3) single field survey sampling effort adjusted systematically for patch area or explicitly validated for level of completeness; (4) single survey with limited effort adjustment or validation; or multiple surveys without adjustment of spatial effort to patch size (see Online resource 4).

### Graphical interpretation

SLOSS analysis was used to infer the effects of subdivision, plotting QH type curves for each dataset and assigning each to one of three exclusive categories as proposed by Fahrig (2017), where the impacts of subdivision were assumed to be: positive, negative or to have no effect based on a curve-overlap criterion (see Online resource 1). As overlap must be compared over a shared range in accumulated area (Online resource 1), this precluded 38 meta-communities where the largest patch was more than 50% of the total combined area, leaving a sample size of 164 (82%) for graphical SLOSS analysis.

### Null model simulations

For the null model approach, I generated 1000 randomized matrices for each of the datasets and for each of these simulated meta-communities, I re-calculated the size-ordered species accumulation curves, producing a 95% simulation interval in species accumulation for each combination of patches. I used a fixed–fixed (FF) null model algorithm, which preserves row and column marginal totals to represent a passive sampling expectation within the constraints of the available presence-absence data. While the FF approach does not strictly result in random matrices, a proportional–proportional algorithm was deemed unreasonable, as it allows row and column totals to vary (Ulrich and Gotelli 2012), thus relaxing the critical area-driven constraint on local scale species richness and the likelihood of observing a species within a patch (i.e., the frequency of occupancy across sites), required for the passive sampling expectation. Moreover, FF algorithms have the benefit of being least sensitive to total species richness and are thus the most appropriate null models when testing patterns of species co-occurrence in comparing matrices that differ in dimensions as was the case here (Ulrich et al. 2018). I used the sequential ‘curveball’ algorithm (Strona et al. 2014), with thinning set to 100. Simulated communities were created using R Package vegan (Oksanen et al. 2020) and all R code is provided as Online resource 5.

### Statistical tests

#### Quantifying effect size

Observed vs expected species accumulation under the null model were analyzed in two ways. I first calculated an effect size for each small-to-large and large-to-small ordering of sites in each meta-community using a mean residual deviation (RD) statistic according to:

1 $$ {\text{RD}} = \frac{1}{{\left( {m - 2} \right)}}\mathop \sum \limits_{i = 2}^{m - 1} \left( {{\text{Obs}}_{{\text{i}}} - {\text{Exp}}_{{\text{i}}} } \right)/{\text{Exp}}_{{\text{i}}} $$

where *m* is the total number of patches in the meta-community, *i* is a valid point of comparison on the accumulation curve (i.e., precluding the first and last patches, which are fixed in the null model algorithm, i.e., *i* = 2, 3, …, *m-*1), Obs_i_ is the observed number of species accumulated in the *i* sites and Exp_i_ is the mean of the simulated communities for the same number of sites, approximating the expectation for passive sampling. The expectation for passive sampling gives the number of species that would be accumulated based on the observed occupancy across all sites. The RD statistic then gives a measure of deviation from this expectation over the entire range of accumulation for each curve individually, where negative values indicate fewer species accumulated than expected according to the null model. As a measure of overall effect size for patch size dependence in species accumulation I used the arithmetic difference in mean RD between small–large and large–small order (i.e., $\Delta {\text{RD}} = \overline{{{\text{RD}}}}_{{{\text{SL}}}} - \overline{{{\text{RD}}}}_{{{\text{LS}}}}$). More negative values indicate greater impact on species accumulation in small–large order (i.e., a positive disproportionate effect relative to passive sampling for large patches), positive values the opposite. The ΔRD statistic quantifies the effect size when ignoring patch size dependence in occupancy. If a total of *X* ha of habitat was protected, one would expect a proportional difference of ΔRD in the species conserved if the *X* ha comprised only the smallest patches than if it comprised the largest patches, where the expectation for the number of species conserved is the passive sampling expectation for *X* ha of habitat. I tested whether the difference in RD differed from zero in either direction across all meta-communities and within habitat types and taxonomic groups using a paired *t* test assuming unequal variance. The ΔRD statistic was compared between levels of meta-community type and taxonomic group using Kruskal–Wallis tests.

#### Testing the frequency of null and alternative hypotheses to passive sampling

In addition to an overall effect size as described in the previous section, the frequency of meeting or exceeding passive sampling in both directions was also analyzed to provide a point of comparison with SLOSS analysis categories. The 95% simulation envelope for both species accumulation curves was used to test the null hypothesis of passive sampling in both directions (i.e., small–large and large–small). For each dataset, comparison of observed and expected species accumulation in both small–large and large–small order had four possible outcomes: 1. observed = expected (O = E), where all observations were within the range of simulations; 2. observed accumulation was greater than expected (O > E), where one or more points were above the range of simulations; 3. observed accumulation was less than expected (O < E) where one or more points fell below the range of simulations; or, 4. one or more points fell both above and below the range of simulations (O <  > E). For each meta-community, this yielded 16 possible mutually exclusive logical conditions combining the two size-ordered simulations (see Online resource 6 for details). If all observed points fell within the simulation envelope in both small–large and large–small order, there was no evidence to reject the null hypothesis that species accumulation was consistent with passive sampling. This provides no evidence of patch size dependence in community assembly (patch size independence, *H*_0_).

However, if observed species accumulation fell either above or below the simulation range, this was interpreted as a rejection of the null hypothesis of passive sampling at the 5% level. Depending on the nature of the deviation from passive sampling, three alternative hypotheses were defined, two of which suggested a disproportionate (relative to passive sampling) effect of patch size (larger or smaller) on the composition of species accumulated. Different combinations of the 16 possible logical states were used to construct the three alternative hypotheses as follows (Online resource 6): If at least one point in the observed accumulation curve fell above the upper 95% limit in large–small order, but all points in small–large order fell either within or below the lower 95% limit in small–large order, or if large–small fell within the passive sampling expectation, but small–large fell below, this was taken as evidence in favor of greater species accumulation in large patches. This result is consistent with the hypothesis that some species preferentially (or only) occupy larger patches (hereafter large patch dependence; alternative hypothesis 1: *H*_AL_). The opposite logical states (small–large only above; large–small within or below; small–large within and large–small below) was taken as evidence in favor of greater species accumulation in small patches—consistent with the hypothesis that some species preferentially (or only) occupy small patches (small-patch dependence; alternative hypothesis 2: *H*_AS_). Other logical states (e.g., at least one data point falls above and below, points only fell below in both directions, etc.) were grouped as a third alternative hypothesis that was inconclusive about patch size dependence. Such combinations occurred in fewer than 9% of meta-communities (Table S6.1, Online resource 6), which were precluded from further analysis. Comparisons relating to the frequency of large or small patch contributions to species accumulation between null models (*n* = 184) and SLOSS analysis (*n* = 164) were therefore based on proportional responses to different metacommunities but remained qualitatively identical when restricted to the 148 metacommunities common to both methods.

#### Post hoc tests of patch size dependence among metacommunities and taxonomic groups

Grouping the metacommunities according to their support for the 3 competing hypotheses for patch size dependence (*H*_0_ = passive sampling or patch size independence, *H*_AL_ = large patch dependence and *H*_AS_ = small patch dependence), I tested for differences in the frequency of patch types (archipelagos, habitat islands and fragments), and broad taxonomic groups (invertebrates, plants, non-avian vertebrates and birds) using Fisher’s Exact Test of proportions. Pairwise post hoc tests were done for significantly different results (*P* <  ~ 0.05) to identify conditions that were more frequent among levels of each factor. Type I error probabilities in each test were adjusted using sequential Bonferroni correction (aka Holm’s method).

#### Sensitivity to covariates

Finally, I tested the sensitivity of findings for both SLOSS analysis and null models to matrix dimensions (number of sites and species), the exponent of the power law island species–area relationship and nestedness on a gradient of patch area. For all metacommunities, I calculated the island species–area relationship exponent in arithmetic space using non-linear least squares regression. I compared the distribution of the exponents among SLOSS analysis and null model patch size dependence classes using Kruskal–Wallis tests to meet distributional assumptions. I was interested in nested subsets because of its relationship to the SLOSS debate, where one would intuitively expect significant nestedness should favor a large patch dependence in species accumulation (Patterson and Atmar 1986). To quantify nestedness on a gradient of patch area I used the NODF metric (Almeida-Neto et al. 2008) calculating a standardized effect size (SES) with the simulated null model communities described in “Null model simulations”. The distribution of values among the SLOSS analysis and null model patch size dependence classes for each of the covariates was tested using Kruskal–Wallis tests. Post hoc tests for differences between factor levels were identified using Dunn’s pairwise rank test with sequential Bonferroni adjustment. All simulations and statistical analyses were done using R 4.0.1 (R Core Team 2020).

## Results

### Evidence for passive sampling vs. alternative hypotheses

**Fig. 2:**
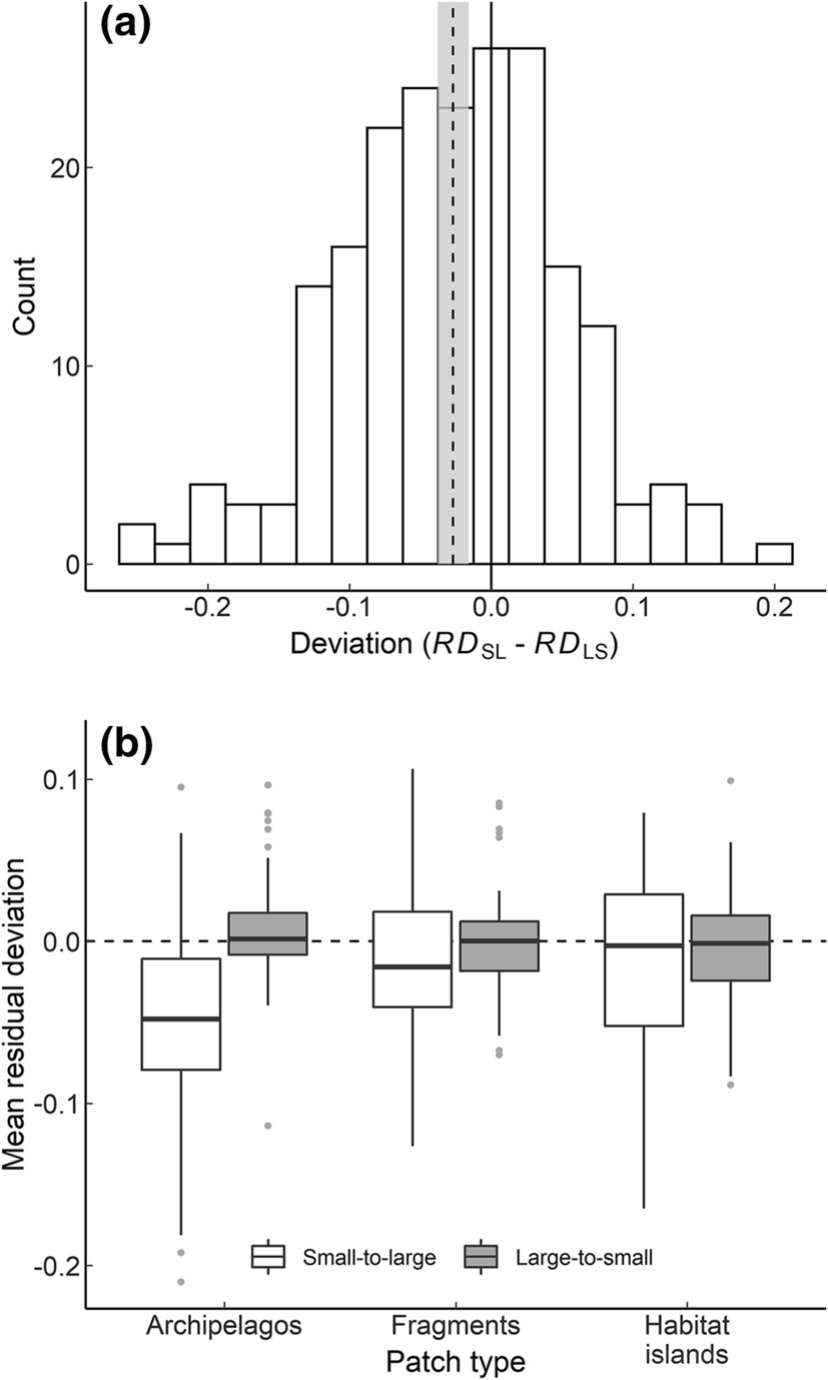
Patch-size dependence in species accumulation **a** difference between observed data and a passive sampling model assuming species have no patch size dependence (Δ*RD*) for all metacommunities (*n* = 202) and **b** individual small–large and large–small curve differences from passive sampling (*RD*) by meta-community type. In both (**a**) and (**b**), negative values mean fewer species were accumulated across the dataset than expected under passive sampling. In (**a**) histogram bars show the number of meta-communities falling within each bin, dashed vertical line shows the mean difference across all datasets (− 0.027) and the gray bar shows the extent of the 95% confidence intervals in this value. In (**b**), boxes show the interquartile range with the median value shown in bold. Hinges show 1.5 times interquartile range

Across all metacommunities, the mean difference from passive sampling in species accumulation was more negative in small–large than large–small comparisons (Fig. 2; Δ*RD* [95% confidence interval] = − 0.027 [− 0.038, − 0.016]; *t* = − 4.86, *df* = 201, *P* < 0.001). Patch-size independence could not be rejected for 40% of metacommunities, while 33% were consistent with large patch dependence (H_AL_) and 18% with small-patch dependence (*H*_AS_). The remainder (18 metacommunities, 8.9%) had no clear response (Table S6.1, Online resource 6). In comparison, SLOSS analysis found no patch size dependence (i.e., overlapping curves) in 25.8% of metacommunities, large patch dependence (i.e., a negative inferred effect of subdivision) in 7.4% of metacommunities and small-patch dependence (i.e., a positive inferred effect of subdivision) in 66.9% metacommunities (Table S7.1, Online resource 7).

**Fig. 3:**
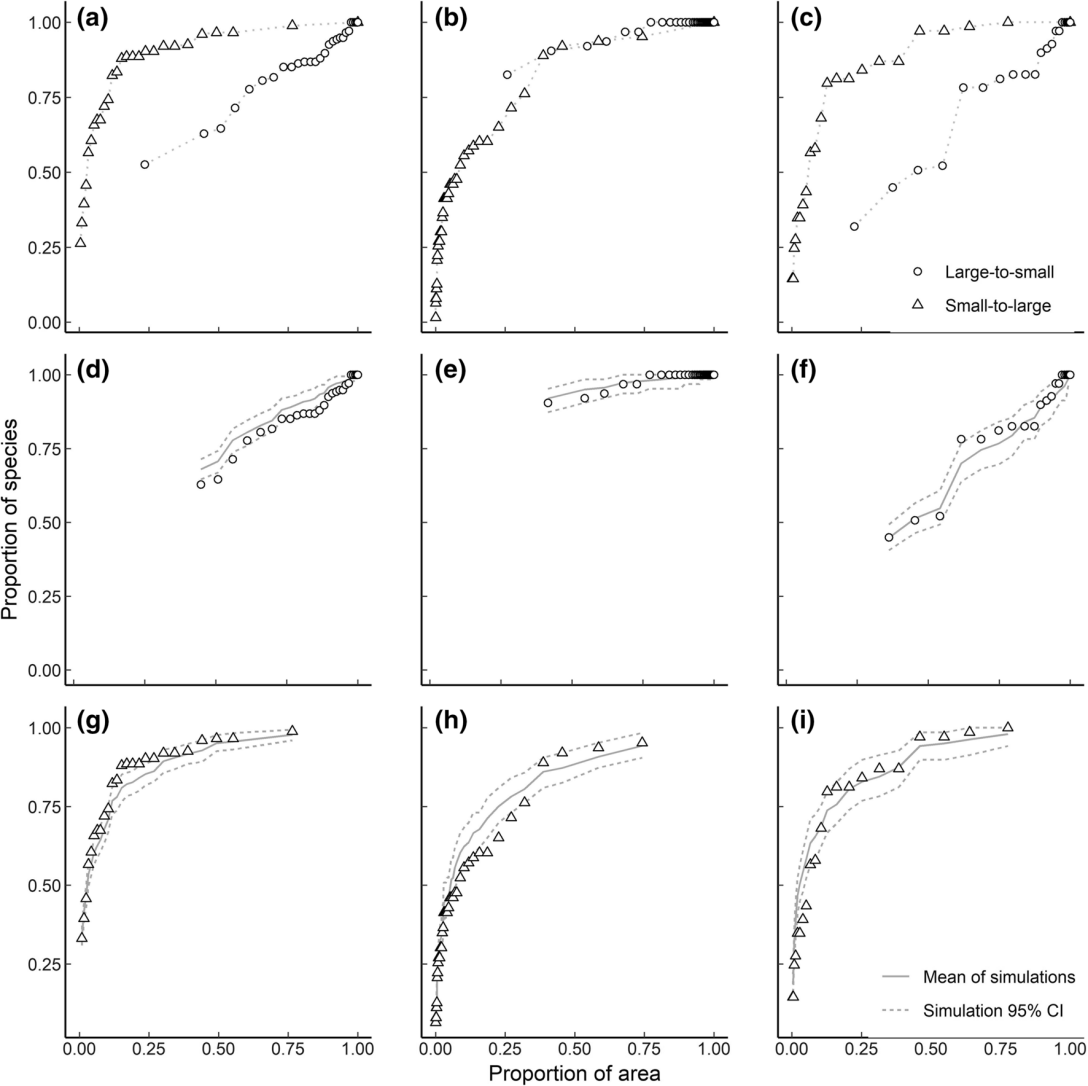
Comparison of inference from SLOSS analysis (top row) and null model simulations (center row, bottom row) for three datasets (columns). Top row (panels **a**–**c**) shows SLOSS analysis. Middle (**d**–**f**) and bottom (**g**–**i**) rows are null model results for large–small and small–large order, respectively. Left column, shows birds on an Australian archipelago (Gibson et al. 2017). Centre column, birds in a Finnish archipelago (Haila et al. 1983). Right column, lizards in Western Australian reserves (Kitchener et al. 1980)

Inconsistent inference on patch size dependence between SLOSS analysis and null models is illustrated for three datasets (Fig. 3). Here, SLOSS analysis suggests small-patch dependence in two datasets (Fig. 3a, c) and no effect of patch size in a third (Fig. 3b). Null models support this conclusion for the first dataset, as large–small accumulation falls only below, while small–large order exceeds 95% simulation intervals (Fig. 3d, g respectively). However, small–large order falls below the lower simulation interval for the second dataset (Fig. 3h) suggesting large patches were important for some species, not evident from SLOSS analysis. The opposite conclusion arises for the third dataset, where large–small order exceeds the simulation interval while small–large falls only below it (Fig. 3f, i).

### Patch-size dependence in metacommunities and taxa from null models and SLOSS analysis

Mean deviations from passive sampling differed among metacommunity types (*χ*^2^ = 9.7, *df* = 2, *P* = 0.008; Fig. 2b; Online resource 7) with archipelagos (Δ*RD*_Arch_ = − 0.050 [− 0.069, − 0.031]) having a more negative median residual deviation (i.e., greater large patch dependence) than either fragments (Δ*RD*_Frag_ = − 0.015 [− 3.0e-2, − 1.5e-05]; Dunn’s pairwise rank test: *z* = 2.76, *P*_adj_ = 0.012) or habitat islands (Δ*RD*_Hab_ = − 0.011 [− 0.031, 0.012]; rank test: *z* = 3.03, *P*_adj_ = 0.007). There was no evidence of any difference in residual deviation between fragments and habitat islands (*P*_adj_ = 0.64), nor among taxonomic groups (KW *χ*^2^ = 2.4, *df* = 3, *P* = 0.49).

Frequency of support for the null and alternative patch size dependence hypotheses also differed between metacommunity types (*P* = 0.007), varying consistently with Δ*RD*, where fragmented landscapes had a higher proportion of metacommunities following passive sampling than archipelagos (60 vs 40% respectively) and a lower proportion of metacommunities with large patch dependence (*H*_AL frag_ = 25%, *H*_AS frag_ = 16% of metacommunities), with archipelagos again more frequently consistent with large patch dependence (i.e., *H*_AL_; *P*_adj_ = 0.012). Differences in frequency of support among taxonomic groups were also evident (*P* < 0.001), with non-avian vertebrates more likely to follow passive sampling than small-patch dependence, relative to invertebrates or birds (both *P*_adj_ < 0.01).

### Sensitivity of SLOSS analysis and null model outcomes to covariates

**Fig. 4:**
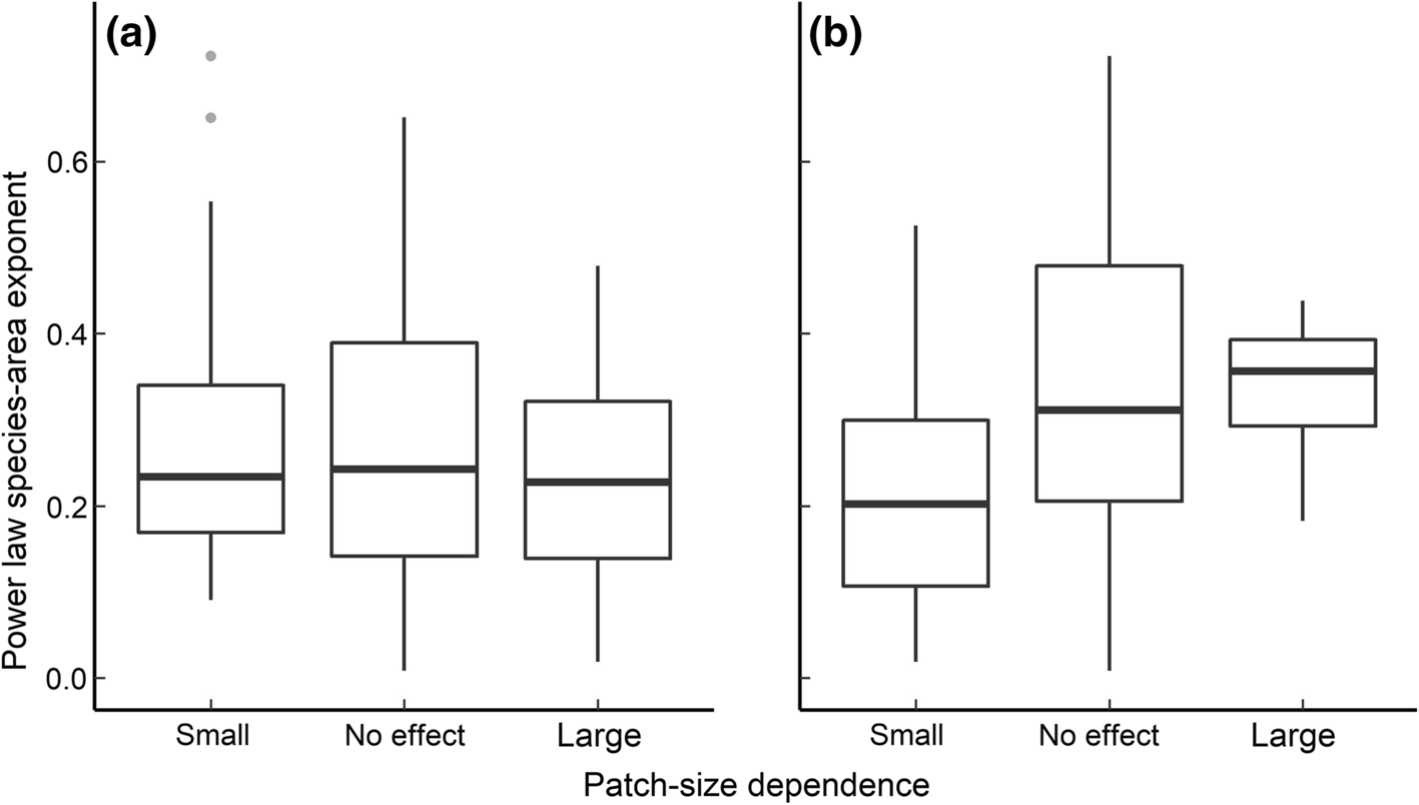
Relationships between the outcomes of **a** null model simulations and **b** SLOSS analysis with the island species–area relationship characterized using the power-law exponent (i.e., *z* value). **a** distribution of exponent values between meta-communities classified according to patch size dependence relative to the passive sampling null model, **b** same values for patch size dependence categories inferred from SLOSS analysis

The exponent of the power law species–area relationship was associated with the outcome of SLOSS analysis (Fig. 4; Kruskal–Wallis *χ*^2^ = 0.42, *df* = 2, *P* < 0.001) but not null model simulations (*P* = 0.28). Lower exponent values (mean ± SD = 0.15 ± 0.13), were associated with a positive effect of subdivision (small-patch dependence) according to SLOSS analysis, larger values (0.42 ± 0.25) were more likely to infer large patch dependence. The value of the exponent in overlapping curves was intermediate to this (0.32 ± 0.18). The outcome of SLOSS analysis did not depend on the residual deviation from passive sampling (*χ*^2^ = 0.92, *df* = 2, *P* = 0.63) but, as would be expected, the most supported hypothesis from null model simulations was strongly dependent (*χ*^2^ = 98.4, *df* = 2, *P* < 0.001).

Statistically significant (*P* < 0.05 for standardized effect size, SES) nestedness on a gradient of patch area occurred in 6.9% of metacommunities, while the opposite pattern (i.e., composition being less nested than expected aka anti-nestedness) occurred in 15.4% of metacommunities. Nestedness on a gradient of patch area was influential on simulation results (*P* = 0.016), where more negative SES (i.e., anti-nestedness) was associated with small-patch dependence (*H*_AS_) relative to metacommunities with either passive sampling (*H*_0_, *P*_adj_ = 0.014) or large patch dependence (*H*_AL_, *P*_adj_ = 0.045). Nestedness on a gradient of patch area did not vary among metacommunities following different SLOSS analysis outcomes (*P* = 0.26).

For null model simulations, mean residual deviation (*RD* statistic) was not affected by the number of patches (Pearson’s *r* = − 0.05, *P* = 0.20) but was negatively correlated with the (log) number of species (*r* = − 0.21, *P* = 0.005). In frequency tests, passive sampling was more likely among metacommunities with fewer patches and fewer total species (both *P* < 0.001). There was only marginal evidence the outcome of SLOSS analysis was associated with the number of patches (*P* = 0.075), but there was a similarly strong dependence on the total number of species (*P* < 0.001). There was also marginal evidence that patch size class depended on the confidence level in the data (*P* = 0.086), but no difference associated with the outcomes in the minimum or maximum confidence levels (i.e., best and worst quality data; *P* = 0.92), nor in the distribution of the *RD* statistic (median: KW *χ*^2^ = 5.5, *df* = 3, *P* = 0.14; variance: Bartlett’s test: *B* = 4.6, *df* = 3, *P* = 0.21). The outcome of SLOSS analysis was sensitive to the level of confidence in the data (*P* = 0.009), and in this case, the lowest confidence datasets were more likely than the highest confidence to return a positive subdivision effect (i.e., small-patch dependence; *P*_adj_ = 0.013).

## Discussion

Conflicting evidence on the biodiversity value of small patches has produced ongoing debate, particularly in the context of managing fragmented habitat (e.g., Fahrig et al. 2019; Fletcher et al. 2018). This study offers two insights. First, it shows that the evidence of relative patch size importance in species accumulation from SLOSS analysis is unreliable and largely a function of the island species–area relationship. More importantly, it shows that when SLOSS-type species accumulation curves are compared against a suitable null model benchmark, small patches are typically not of equal habitat value for all species in the landscape (Blake and Karr 1984; Matthews et al. 2014). This can be true even where they support greater richness for a given total area.

### Implications for fragmented habitat

The relative importance of small and large patches for species representation is of most conservation importance in fragmented landscapes. Based on the overall negative mean residual deviation statistic in this study, preferential protection of larger fragments would be expected to provide habitat suitable for a greater proportion of species in the landscape. This might warrant a decision to protect large patches over small ones in spite of greater richness in the latter (Fahrig et al. 2021). However, it is not that straightforward in practice, as small-patch dependence was inferred here in almost one in six fragmented meta-communities and the loss of small patches from fragmented landscapes could have serious consequences for extant native diversity (Deane and He 2018; Wintle et al. 2019). This supports a view where habitat patches of all sizes are valued (Deane and He 2018; Rösch et al. 2015; Wintle et al. 2019) but, in general, the more area habitat patches represent both individually (Chase et al. 2020; Haddad et al. 2015; Matthews et al. 2014) and collectively (Andrén 1994; Fahrig 2013; Watling et al. 2020), the better the likely outcome for representation of all species.

The effect size for fragmented meta-communities was small, but there could be several reasons, it represents a lower limit. It is possible that the landscapes analyzed have not yet reached equilibrium, in which case the small negative effect size could partly reflect an unpaid extinction debt in small patches (Tilman et al. 1994). Other ecological considerations also might reduce the observed effect size, for example, perhaps species dependent upon larger patches were rapidly lost following fragmentation (Gibson et al. 2013) or the contrast between habitat and the surrounding matrix was not pronounced, reducing the impacts of fragmentation on the taxa concerned (Laurance et al. 2011). Data also offer a likely source of underestimation of effect size.

### Uncertainties

Undoubtedly the major uncertainty in this analysis are the data. The problem is common to both methods and must be taken into account when considering these results. Not only do presence–absence data offer limited power to infer diversity effects in varying size habitats (Chase et al. 2019; Haila and Hanski 1984), more important is the difficulty in ensuring species lists for each patch represents a complete census. A negative relationship between sampling effort and patch size for these types of data appears almost ubiquitous (Deane et al. 2020), increasing the probability of finding small patch dependence in SLOSS comparisons (Deane et al. 2020; Fahrig 2020; Results). Even though null model analysis did not show any clear relationship with data confidence levels, only 5% of the data from fragments were of the highest confidence, compared with 45% of archipelago datasets. Excluding data of the lowest confidence increased the observed effect size for fragments by almost 50% (Δ*RD*_Frag_ = − 0.022 vs − 0.015 using all data). It seems probable that the effect sizes and patterns of patch size dependence identified from null models would more likely understate the importance of larger patches for species representation.

### Large-patch dependence greater for archipelago biota than fragments or habitat islands

Archipelagos were more likely to show large patch dependence than either fragments of formerly continuous habitat or naturally occurring habitat islands, consistent with patch-scale evidence (Chase et al. 2020; Gooriah et al. 2021). Post hoc analysis of null model results showed an interesting numerical trend consistent with island biogeography (MacArthur and Wilson 1967), where increasingly isolated archipelagos had increasing large patch dependence (Δ*RD* for reservoir or freshwater lake islands = − 0.035, continental archipelagos = − 0.050, oceanic archipelagos = − 0.078). For null models, the frequency of large patch dependence in oceanic island metacommunities was 64%, while SLOSS analysis suggested the opposite, with none showing large patch dependence and 66% having small-patch dependence (see Table S7.1, Online resource 7, for other frequency comparisons).

Habitat islands presented the least evidence of any large patch dependence and were the only metacommunity type to have a positive (albeit not statistically different from zero) median *RD*, suggesting relatively limited patch size dependence*.* These results suggest that of all metacommunity types examined, small habitat islands (particularly ponds and wetlands) are most likely to make important contributions to landscape species representation, which has often been reported (Deane et al. 2017; Flinn et al. 2008; Oertli et al. 2002; Peintinger et al. 2003; Richardson et al. 2015). Most fragmented landscapes also contain such naturally discrete habitats, subject to similar land use impacts and habitat loss, which warrant conservation effort.

In contrast with patch type, effect size did not differ between taxonomic groups. This might be in part due to the coarse nature of the classifications used but is not an unusual result (Chase et al. 2020; Gooriah et al. 2021). There was, however, weak evidence invertebrates and birds had a higher frequency of small-patch dependence than non-avian vertebrates. For invertebrates, this is consistent with prior work (Deane and He 2018; Rösch et al. 2015; Tscharntke et al. 2002) but evidence for birds is equivocal, particularly for habitat specialists (Blake and Karr 1984; Carrara et al. 2015; Matthews et al. 2014). In contrast, failure to reject passive sampling for a higher proportion of non-avian vertebrate metacommunities is consistent with expectation and likely reflects their greater area dependence and rapid decline to local extinction in isolated small patches (Bolger et al. 1997; Gibson et al. 2013).

### SLOSS analysis is not a reliable approach

Even if problems with scale dependence in quantifying species richness are overlooked, it is clear from this analysis that QH curves can contribute nothing to our understanding of subdivision effects. SLOSS analysis outcomes are unrelated to whether species form nested subsets on a gradient of patch area (Mac Nally and Lake 1999; Results), which is counter-intuitive and the opposite of expectation (Tjørve 2010; Worthen 1996). Moreover, the outcome depends on the island species–area relationship (ISAR), which describes how species richness changes with increasing patch area in independent draws from the regional species pool (Scheiner et al. 2011) not how species accumulate as patches are combined (Matthews et al. 2016). A relationship between SLOSS analysis and the ISAR was recently noted for multiple taxa in island archipelagos (Liu et al. 2022); this study explains why it is a general result. This relationship with the ISAR also explains how SLOSS analysis constrained only to specialist or endangered species might still show small patch preference (e.g., Fahrig 2020; Richardson et al. 2015; Riva and Fahrig 2022; Tscharntke et al. 2002); any taxon with an island species–area curve power law exponent less than ~ 0.3 is likely to result in QH curves where the small–large curve lies always above the large–small curve.

The difference in conclusions between SLOSS analysis and null models highlights the need to control for sampling effects when analyzing richness scaling patterns in discrete habitat patches of irregular size (Chase et al. 2019; Chase et al. 2018). Both geometric effects (Deane et al. 2022; Fig. 1; Kobayashi 1985; May et al. 2019) and increased beta diversity among smaller patches (Deane et al. 2020; Fahrig 2020; Liu et al. 2018; MacDonald et al. 2018b) promote greater richness in subdivided habitat. This should be the expectation and methods should explicitly account for this. Ultimately, this study highlights the need for high quality abundance data, within-patch replication of standard sized samples and appropriate statistical methods to correctly interpret the scaling of species richness with area and to understand its mechanistic origins (Chase et al. 2019, 2018; Hill et al. 1994).

## Supplementary Information

Below is the link to the electronic supplementary material.

Supplementary file1 (DOCX 307 KB)

Supplementary file2 (XLSX 63 KB)

Supplementary file3 (TXT 13 KB)

Supplementary file4 (DOCX 26 KB)

## Data Availability

All data used in this study are in the public domain (see Online resource 3 for data sources) and the results of analyses are provided in Online resource 4.

## Code availability

R code provided in Online resource 5.
