# Supplementary material for: Species accumulation in small–large vs large–small order: more species but not all species?
Source: Oecologia. 2022 Sep 17;200(1-2):273–84. doi: 10.1007/s00442-022-05261-1 (PMC9547801; doi:10.1007/s00442-022-05261-1)
Supplement: Supplementary file 1 — Supplementary file1 (DOCX 307 KB) [file 442_2022_5261_MOESM1_ESM.docx]

**Species accumulation in small-large vs large-small order: more species but not all species?**

DC Deane

# Online resource 1. Appendix S1 - Prior criticism and inferential pitfalls in QH curves

Prior criticism of the use of Quinn-Harrison (QH) curves for inference can be divided into those relating to how data are collected and those associated with how the data are analysed. Gavish et al. (2012) pointed out problems associated with sampling patches of different size, which confounds sampling effort with patch size. Developing statistical techniques to objectively assign a difference between the two size-ordered curves, has proven even more problematic. The original saturation index metric proposed at the time of introducing the graphical method (Quinn and Harrison 1988) was statistically biased (Ramsey 1989), leading Mac Nally and Lake (1999), to propose a new index, η, which incorporated information on species occupancy, weighting rarer species higher. Mac Nally and Lake also proposed testing for a statistical difference using a Monte Carlo approach, a clear improvement on the original methods. While the η statistic has not been widely applied, QH curves remain popular for both graphical purposes and inference.

However, there are other subtle problems with the use of the curves for inference based on graphical interpretation. Accumulating patch area vectors in the reverse size-order rarely (if ever) leads to coinciding incremental values. In QH curves then, comparing species accumulation for a common area thereby necessitates some form of interpolation between the data points. This is invariably linear, despite overwhelming evidence that species accumulation within patches is a decelerating function of area (e.g., a power function). Ramsey (1989), first identified the issue of interpolation, where connecting the curve from the origin to the first patch represents only within, not among patch species accumulation. The same applies when any two patches are combined, but particularly when connecting the last patch in small-to-large order to the point representing total area and species number. As a result, an appropriate window for graphical comparisons based on overlap in curves should include only points on either accumulation curve falling within the interval [*A*_SL,_ *A*_TOT_ – *A*_SL_], where *A*_SL_ is the area of the single largest patch; and, *A*_TOT_ is the total area of all patches.

The decision to adopt linear interpolation potentially has other consequences for graphical comparisons and to illustrate this we can plot accumulation in a stepwise manner, consistent with the known information (Fig. S1.1). In some cases, this decision could affect the outcome of graphical comparison of overlap (e.g., Fig. S1.1A and B). In practice, QH curves do not often present a pattern where this difference would change conclusions, but this helps illustrate the complexity of basing inference on a graphical interpretation.


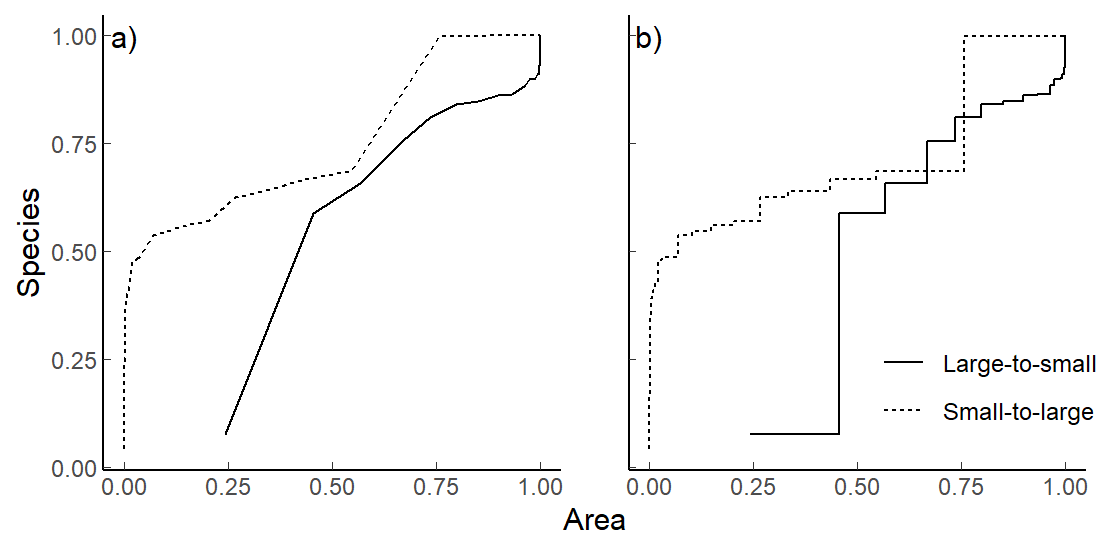


1. QH curves and uncertainty in inference based on overlap of size-ordered species accumulation curves. When smooth curves are compared, this assumes a linear interpolation between points in the accumulation as shown in a), a typical pair of QH curves, with data points omitted for clarity. However, these data do not truly provide any information about scaling of diversity with area within patches and, arguably, should therefore be b) conceptualised in a stepwise manner. For these data, representing ferns in bioregions of sub-Saharan Africa (Aldasoro et al. 2004) ), the conclusion regarding fragmentation effects would change from positive to no effect.

Recently, Fahrig (2017; 2020) has used an overlap criterion to infer the effects of subdivision on species richness using QH curves (often called ‘SLOSS analysis’). The criterion interprets accumulation curves that do not cross as evidence of an effect of subdivision, with the inferred direction of impact on species richness of groups of patches (positive or negative) dependent on their relative positions (Fig S1.2). Criticism of this graphical re-interpretation of the curves in terms of the response of diversity to subdivision was raised by Fletcher et al. (2018). However, that objection was based on an earlier critique by Mac Nally and Lake (1999), focussed on the original metric proposed by Quinn and Harrison (1988), which is not used in SLOSS analysis. There is, however, a more fundamental question arising from comparing species accumulation among sites in reverse size order, which is illustrated in Appendix S2.


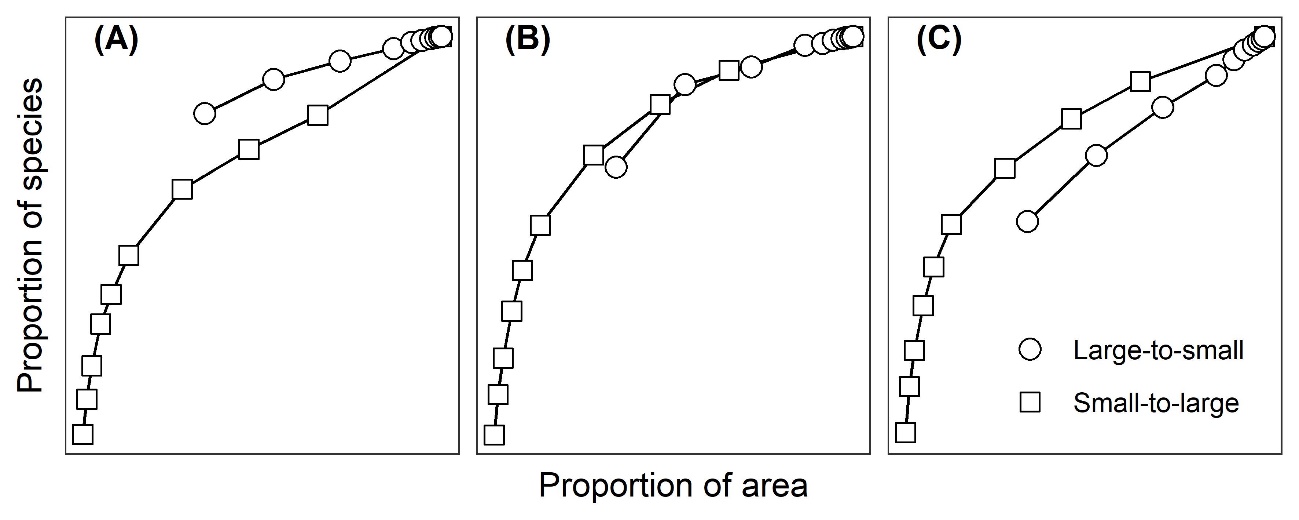


1. The basis for inference based on SLOSS analysis. The *x*-axis shows the accumulated area as patches are combined in decreasing (large-small) and increasing (small-large) size order and the number of species accumulated is shown on the *y*-axis. (A) If the large-small curve lies always above the small-large curve, a negative effect of subdivision is inferred; (B) where the curves overlap, there is no apparent effect; and (C) when the small-large curve is above the large-small curve, a positive effect of subdivision is inferred. Adapted from Fahrig (2017)

# Online resource 2: Appendix S2 - Effect of size ordering on probability of encountering species

To illustrate the effect of combining patches in order of their size on the probability of encountering new species, consider a group of three patches that follow a typical island species–area relationship, two small and one large (Fig. S2.1), and how likely it is that we will encounter new species as we add each patch. This can be quantified using the beta partition: β = (1 - ᾱ/γ), where ᾱ (mean alpha diversity) is the average number of species present at local scale (i.e., among 2 or more accumulated patches) and γ (gamma diversity) is the total species number observed in all samples. Based on the multiplicative partition of beta diversity proposed by Whittaker (1960) the beta partition represents the probability that a species present in the region (or in this case, the dataset) is absent from a local sample. The higher this probability, the more likely new species are encountered as the patch is added. Notice how the order of accumulation affects this probability for the middle patch in a simple three patch system following a power law island species-area relationship (patch B, Fig S2.1), where the probability is higher when combining sites in small-large order than the reverse. The same area of habitat has been added, but a greater probability of adding new species results from combining in small-large order. This calculation can be extended over a more realistic patch network to create a ‘cumulative beta partition’ curve (Fig. S2.2). This shows that the probability of encountering new species is greater for every patch - regardless of area - when combining patches in small-large order. It is necessary to control for these passive sampling effects using a suitable null model (Chase et al. 2019).


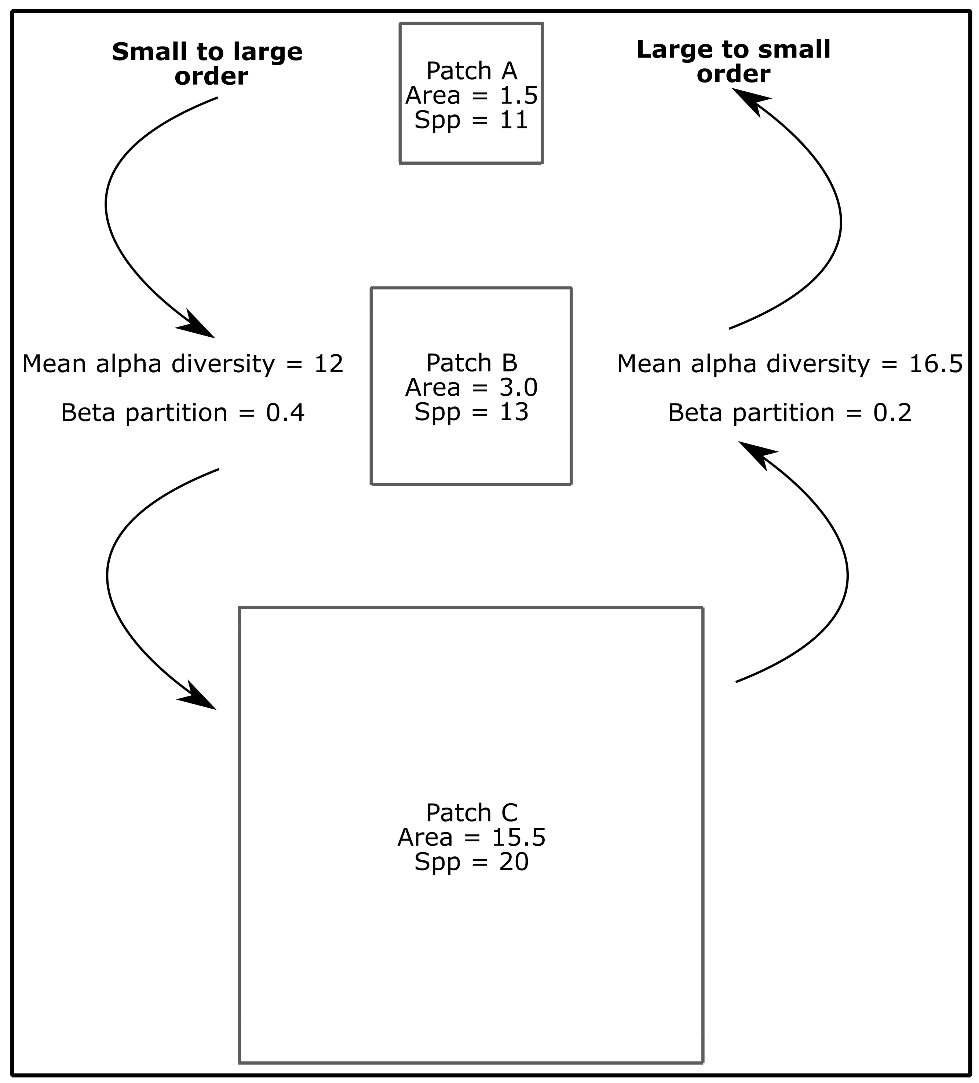


1. The effect of combining patches in increasing and decreasing size order on the expected probability of encountering a new species as that patch is added (quantified as the beta partition β = (1 - ᾱ/γ)). Each square represents a patch of different size, with the number of species in each patch and overall species for all patches predicted from a power law species-area relationship *Species* = 10**Area*^0.25^. Gamma diversity for this illustration was calculated from the SAR using the total area of all patches.


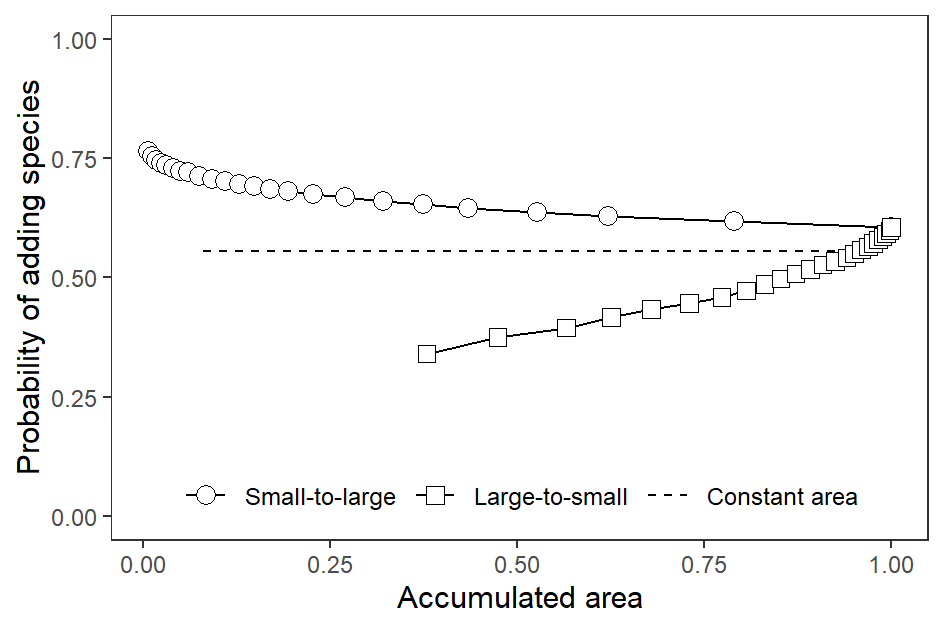


1. Extending the calculation in Fig. S2.1 over a distribution of patch sizes representative of published data shows the probability of encountering a new species for every patch (quantified as the beta partition) is higher under small-large size order (open circles) than large-small order (open squares). If, as is usually the case in research, accumulation curves are built for patches of uniform size, expected alpha diversity is the same for each patch and the expected probability of adding a new species as patches are accumulated is constant (dashed line). Here patch area was simulated using a lognormal distribution (see below for justification), and the beta partition was calculated assuming a power law species-area relationship as per Fig. S2.1.

## The SLOSS sampling design employed in continuous habitat

Although a SLOSS analysis type sampling design and analytical approach would not typically be used in a continuous landscape, it is illustrative to show the effects of so doing, using stem-mapped forest data (Fig. 1 main text, Fig. S2.3). Although it is possible that any of the three SLOSS analysis outcomes could arise (shown by the width of the 95% sampling intervals in Fig. S2.3), on average, in the four stem-mapped forests analyzed here, the small-large curve lies above that of the large-small curve, consistent with the most common result of SLOSS analysis. Clearly one would not use this evidence to claim that several small patches of forest will preserve more species on average than a single large patch, even though it is numerically true.

This understanding should also be applied in SLOSS studies. If for no other reason than geometric effects of subdivision, greater richness in subdivided habitat is the most appropriate null model. Careful sampling design and analysis is required to control for passive sampling effects in order to distinguish patch-size dependence in the contribution of random and deterministic ecological processes affecting beta diversity. Because of the expectation of higher richness in subdivided habitat, which is further exaggerated by reverse size-ordered combinations (Fig. S2.2), SLOSS analysis is not able to unambiguously distinguish between the effects of sampling design and ecological processes that act in the same direction.


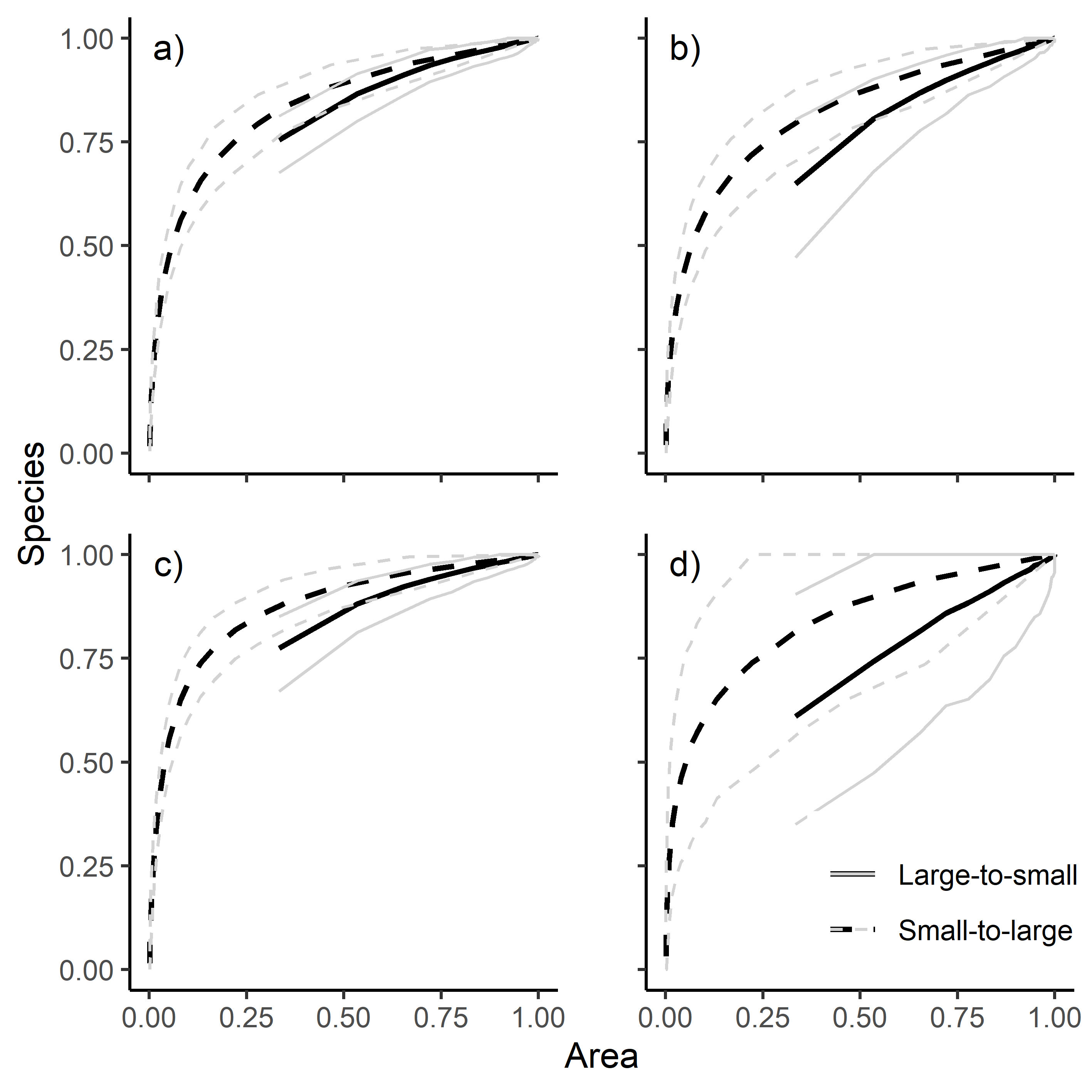


1. SLOSS analysis sampling and analytical design for four continuous stem-mapped forest plots. Results are for 20 quadrats of area drawn from a lognormal distribution and randomly positioned in the forest plot. Black lines show mean result, with grey lines showing 95% sampling intervals from 200 replicates. (a) 50-ha tropical forest, Panama (Barro Colorado Island as per main text) (b), (c) subtropical forest, China (d) temperate forest, North America.

## Simulating the cumulative beta partition curves

To illustrate the effect of reversing patch size order on cumulative beta diversity in the previous section, I first tested the patch size distribution for 202 published datasets (see Appendix S3), using AIC to compare between lognormal and exponential distributions. Over 70% of datasets followed a lognormal distribution. The mean parameter values for all datasets where the lognormal distribution had the most support were: µ = -5.2, σ = 1.7. I used these parameters to simulate a set of 25 habitat patches and adjusted total patch area to 20000 units (note results are qualitatively identical, whatever area units are used). I then assumed patches followed a power law species area relationship (SAR) following $Species=20.{area}^{0.25}$. The SAR then allowed me to estimate the species richness for each simulated patch area, and the total number of species in the total combined area of 20000 units (i.e., gamma diversity). I then calculated a ‘cumulative beta partition’ for the small-to-large accumulation of sites as follows. Although this simulation assumes a nested species-area relationship, provided the island species area relationship is reasonably well described by a power function (say with R^2^ > ~ 0.4), empirical data follow the cumulative partition and the probability of encountering new species is greatest for every patch in small-large order (see Fig. S2.4 for examples).

The first datum was calculated by estimating mean alpha diversity for the two smallest patches. I then calculated the beta partition at this estimate of $\bar{\alpha}$, which was matched to the accumulated area in the two smallest patches. I then added the third smallest site and again calculated $\bar{\alpha}$, allowing an estimate of the beta partition corresponding to the accumulation of three patches. I repeated this process until all patches were combined to provide a cumulative beta partition curve for small-to-large patch ordering and repeated this process combining patches in large-to-small size order. If local samples are all equal in area, we assume this results in an equal number of species as predicted by the SAR, and the cumulative beta partition remains constant as sites are accumulated. Although in nature patches of equal area will differ from the species-area relationship predictions in the number of species they contain, this is not important for illustrating the concepts and the general relationship holds for islands that follow a decelerating species-area relationship (Fig. S2.4).


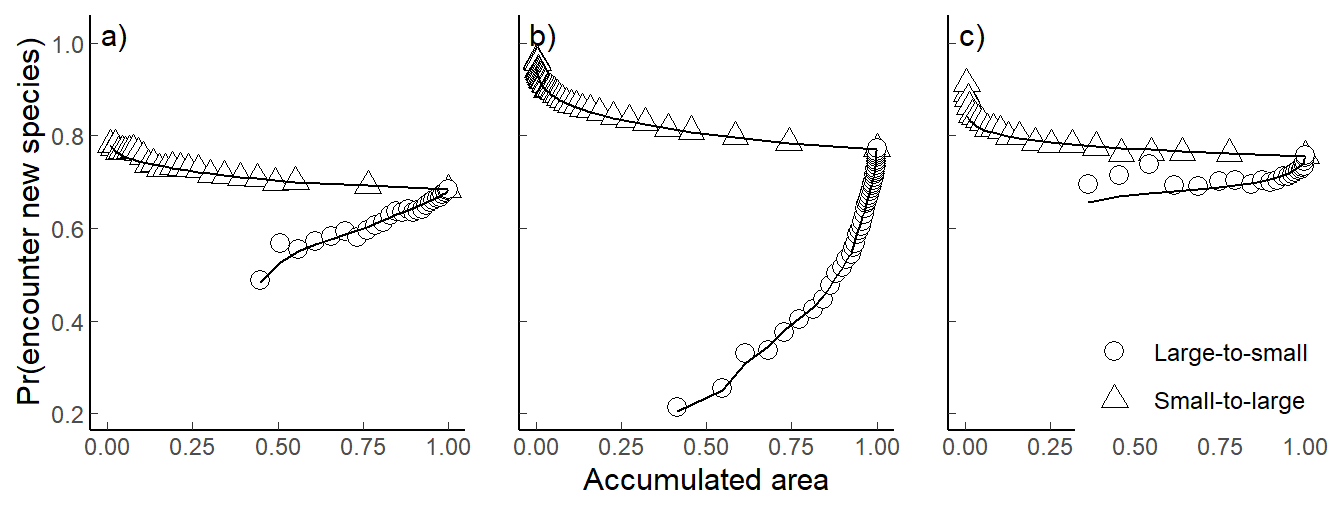


1. Fit of the cumulative beta diversity partition for three empirical datasets. Points show the cumulative probability of encountering a new species as each patch is added in small-large (open triangles) or large-small (open circles) size order. The curves show the modelled cumulative beta partition (as in Fig. S2.2) using the area of the patch and the parameters of the fitted island species area relationship for each dataset. Data as per Fig. 3, main text.

# Online resource 3: Appendix S3 - Data sources

Adler, G.H. & Wilson, M.L. (1985) Small mammals on Massachusetts islands - the use of probability functions in clarifying biogeographic relationships. Oecologia, 66, 178-186.

Aldasoro, J.J., Cabezas, F. & Aedo, C. (2004) Diversity and distribution of ferns in sub-Saharan Africa, Madagascar and some islands of the South Atlantic. Journal of Biogeography, 31, 1579-1604.

Almeida-Gomes, M., Rocha, C.F.D. & Vieira, M.V. (2016) Local and Landscape Factors Driving the Structure of Tropical Anuran Communities: Do Ephemeral Ponds have a Nested Pattern? Biotropica, 48, 365-372.

Almeida-Neto, M., Guimaraes, P., Guimaraes, P.R., Loyola, R.D. & Ulrich, W. (2008) A consistent metric for nestedness analysis in ecological systems: reconciling concept and measurement. Oikos, 117, 1227-1239.

Anciaes, M. & Marini, M.A. (2000) The effects of fragmentation on fluctuating asymmetry in passerine birds of Brazilian tropical forests. Journal of Applied Ecology, 37, 1013-1028.

Anderson, R.S. (1974) Crustacean plankton communities of 340 lakes and ponds in and near national parks of Canadian Rocky Mountains. Journal of the Fisheries Research Board of Canada, 31, 855-&.

Andrade, R.D. & Marini, M.A. (2002) Bird species richness in natural forest patches in southeast Brazil. Lundiana, 3, 141-149.

Antao, L.H., McGill, B., Magurran, A.E., Soares, A. & Dornelas, M. (2019) beta-diversity scaling patterns are consistent across metrics and taxa. Ecography, 42, 1012-1023.

Aranda, R. & Olivier, R.D. (2017) Preliminary study of species-area, isolation and impact of environmental heterogeneity on insect communities in natural patches in the Brazilian Pantanal. Entomological News, 126, 312-327.

Arechavaleta, M., Zurita, N., Marrero, M.C. & Martin, J.L. (2005) Lista preliminary de especies silvestres de Cabo Verde (hongos plantas y animals terrestres). In: (ed. M.O.E.a.S. Planning). Government of the Canaries, Santa Cruz de Tenerife.

Ávila, A.C., Pires, M.M., Rodrigues, E.N.L., Costi, J.A.R., Stenert, C. & Maltchik, L. (2019) Drivers of the beta diversity of spider assemblages in southern Brazilian temporary wetlands. Ecological Entomology,

Azeria, E.T. (2004) Terrestrial bird community patterns on the coralline islands of the Dahlak Archipelago, Red Sea, Eritrea. Global Ecology and Biogeography, 13, 177-187.

Baker, R.J. & Genoways, H.H. (1978) Zoogeography of Antillean bats. Special Publication Academy of Natural Sciences Philadelphia, 53-97.

Báldi, A. & Kisbenedek, T. (1997) Orthopteran assemblages as indicators of grassland naturalness in Hungary. Agriculture Ecosystems & Environment, 66, 121-129.

Báldi, A. & Kisbenedek, T. (1999) Orthopterans in small steppe patches: an investigation for the best-fit model of the species-area curve and evidences for their non-random distribution in the patches. Acta Oecologica-International Journal of Ecology, 20, 125-132.

Ball, O.J.P., Pohe, S.R. & Winterbourn, M.J. (2015) Littoral macroinvertebrate communities of dune lakes in the far north of New Zealand. New Zealand Journal of Marine and Freshwater Research, 49, 192-204.

Baz, A. & Garcia-Boyero, A. (1995) The effects of forest fragmentation on butterfly communities in central Spain. Journal of Biogeography, 22, 129-140.

Baz, A. & Garcia-Boyero, A. (1996) The SLOSS dilemma: a butterfly case study. Biodiversity and Conservation, 5, 493-502.

Behle, W.H. (1978) Avian biogeography of the Great Basin and inter mountain region. Great Basin Naturalist Memoirs, 55-80.

Benedick, S., Hill, J.K., Mustaffa, N., Chey, V.K., Maryati, M., Searle, J.B., Schilthuizen, M. & Hamer, K.C. (2006) Impacts of rain forest fragmentation on butterflies in northern Borneo: species richness, turnover and the value of small fragments. Journal of Applied Ecology, 43, 967-977.

Bengtson, S.A. & Bloch, D. (1983) Island land bird population densities in relation to island size and habitat quality on the Faroe Islands. Oikos, 41, 507-522.

Berglund, H. & Jonsson, B.G. (2003) Nested plant and fungal communities; the importance of area and habitat quality in maximizing species capture in boreal old-growth forests. Biological Conservation, 112, 319-328.

Bittencourt-Silva, G.B. & Silva, H.R. (2014) Effects of Fragmentation and Sea-Level Changes upon Frog Communities of Land-Bridge Islands off the Southeastern Coast of Brazil. Plos One, 9

Blake, J.G. (1991) Nested subsets and the distribution of birds on isolated woodlots. Conservation Biology, 5, 58-66.

Bolger, D.T., Alberts, A.C., Sauvajot, R.M., Potenza, P., McCalvin, C., Tran, D., Mazzoni, S. & Soule, M.E. (1997) Response of rodents to habitat fragmentation in coastal southern California. Ecological Applications, 7, 552-563.

Bond, R.R. (1957) Ecological distribution of breeding birds in the upland forests of southern Wisconsin. Ecological Monographs, 27, 352-384.

Boomsma, J.J., Mabelis, A.A., Verbeek, M.G.M. & Los, E.C. (1987) Insular biogeography and distribution ecology of ants on the Frisian Islands. Journal of Biogeography, 14, 21-37.

Brown, J.H. (1971) Mammals on mountaintops - nonequilibrium insular biogeography. American Naturalist, 105, 467-478.

Brown, J.H. (1978) The theory of insular biogeography and the distribution of boreal birds and mammals. Great Basin Naturalist Memoirs, 209-227.

Burbrink, F.T., McKelvy, A.D., Pyron, R.A. & Myers, E.A. (2015) Predicting community structure in snakes on Eastern Nearctic islands using ecological neutral theory and phylogenetic methods. Proceedings of the Royal Society B-Biological Sciences, 282

Cabrera-Guzman, E. & Reynoso, V.H. (2012) Amphibian and reptile communities of rainforest fragments: minimum patch size to support high richness and abundance. Biodiversity and Conservation, 21, 3243-3265.

Canedoli, C., Manenti, R. & Padoa-Schioppa, E. (2018) Birds biodiversity in urban and periurban forests: environmental determinants at local and landscape scales. Urban Ecosystems, 21, 779-793.

Capizzi, D., Luiselli, L. & Papi, R. (2015) Temporal changes in Mediterranean bird communities across fragmented and continuous forests. Ecological Research, 30, 615-624.

Castelletta, M., Thiollay, J.M. & Sodhi, N.S. (2005) The effects of extreme forest fragmentation on the bird community of Singapore Island. Biological Conservation, 121, 135-155.

Charles, J.K. & Ang, B.B. (2010) Non-volant small mammal community responses to fragmentation of kerangas forests in Brunei Darussalam. Biodiversity and Conservation, 19, 543-561.

Chepinoga, V.V., Zverev, V.E., Zvereva, E.L. & Kozlov, M.V. (2012) Vascular plants on the islands and peninsulas of Maloe More (Lake Baikal): patterns of diversity and species turnover. Boreal Environment Research, 17, 219-236.

Chiarucci, A., Fattorini, S., Foggi, B., Landi, S., Lazzaro, L., Podani, J. & Simberloff, D. (2017) Plant recording across two centuries reveals dramatic changes in species diversity of a Mediterranean archipelago. Scientific Reports, 7

Clark, A.T., Rykken, J.J. & Farrell, B.D. (2011) The Effects of Biogeography on Ant Diversity and Activity on the Boston Harbor Islands, Massachusetts, USA. Plos One, 6

Connor, E.F., Collins, M.D. & Simberloff, D. (2013) The checkered history of checkerboard distributions. Ecology, 94, 2403-2414.

Conroy, C.J., Demboski, J.R. & Cook, J.A. (1999) Mammalian biogeography of the Alexander Archipelago of Alaska: a north temperate nested fauna. Journal of Biogeography, 26, 343-352.

Corti, C., Masseti, M., Delfino, M. & Perez-Mellado, V. (1999) Man and herpetofauna of the Mediterranean islands. Revista Espanola de Herpetologia, 13, 83-100.

Cramer, K.L. (1994) New mammal record for Fremont Island with an updated checklist of mammals on islands in the Great Salt Lake Great Basin Naturalist, 54, 287-289.

Crooks, K.R. (2002) Relative sensitivities of mammalian carnivores to habitat fragmentation. Conservation Biology, 16, 488-502.

Crooks, K.R., Suarez, A.V., Bolger, D.T. & Soule, M.E. (2001) Extinction and colonization of birds on habitat islands. Conservation Biology, 15, 159-172.

Crowe, T.M. (1979) Lots of weeds - insular phytogeography of vacant urban lots. Journal of Biogeography, 6, 169-181.

Crowell, K.L. (1986) A comparison of relict versus equilibrium models for insular mammals of the Gulf of Maine. Biological Journal of the Linnean Society, 28, 37-64.

Daily, G.C. & Ehrlich, P.R. (1995) Preservation of biodiversity in small rain-forest patches - rapid evaluations using butterfly trapping. Biodiversity and Conservation, 4, 35-55.

Daily, G.C., Ehrlich, P.R. & Sanchez-Azofeifa, G.A. (2001) Countryside biogeography: Use of human-dominated habitats by the avifauna of southern Costa Rica. Ecological Applications, 11, 1-13.

Dalecky, A., Chauvet, S., Ringuet, S., Claessens, O., Judas, J., Larue, M. & Cosson, J.F. (2002) Large mammals on small islands: Short term effects of forest fragmentation on the large mammal fauna in French Guiana. Revue D Ecologie-La Terre Et La Vie, 145-164.

Dapporto, L. & Cini, A. (2007) Faunal patterns in Tuscan archipelago butterflies: The dominant influence is recent geography not paleogeography. European Journal of Entomology, 104, 497-503.

Dapporto, L. & Dennis, R.L.H. (2008) Species richness, rarity and endemicity on Italian offshore islands: complementary signals from island-focused and species-focused analyses. Journal of Biogeography, 35, 664-674.

Davidson, R.L., Rykken, J. & Farrell, B. (2011) Carabid beetle diversity and distribution in Boston Harbor Islands national park area (Coleoptera, Carabidae). Zookeys, 497-526.

Davies, R.G., Hernandez, L.M., Eggleton, P., Didham, R.K., Fagan, L.L. & Winchester, N.N. (2003) Environmental and spatial influences upon species composition of a termite assemblage across neotropical forest islands. Journal of Tropical Ecology, 19, 509-524.

Davis, R., Dunford, C. & Lomolino, M.V. (1988) Montane mammals of the American Southwest - the possible influence of post-Pleistocene colonization. Journal of Biogeography, 15, 841-848.

de Castro, E.B.V. & Fernandez, F.A.S. (2004) Determinants of differential extinction vulnerabilities of small mammals in Atlantic forest fragments in Brazil. Biological Conservation, 119, 73-80.

Deane, D. (2016) Fleurieu Peninsula wetlands collated plant survey data Version 2, DOI: 10.4227/05/57450480691AA. Available via ÆKOS Data Portal (Persistent hyperlink: http://www.aekos.org.au/dataset/239810), University of Adelaide.

Deane, D.C. & Walters, A.C. (2008) Baseline survey of refugia pools in the north-eastern Willochra Creek and Western Lake Frome Catchments, DWLBC Report 2008/15. Government of South Australia, Adelaide.

Dennis, R.L.H., Shreeve, T.G., Olivier, A. & Coutsis, J.G. (2000) Contemporary geography dominates butterfly diversity gradients within the Aegean archipelago (Lepidoptera : Papilionoidea, Hesperioidea). Journal of Biogeography, 27, 1365-1383.

Dickman, C.R. (1987) Habitat fragmentation and vertebrate species richness in an urban environment. Journal of Applied Ecology, 24, 337-351.

Dinesen, L., Lehmberg, T., Rahner, M.C. & Fjeldsa, J. (2001) Conservation priorities for the forests of the Udzungwa Mountains, Tanzania, based on primates, duikers and birds. Biological Conservation, 99, 223-236.

Ding, C.Z., Jiang, X.M., Xie, Z.C. & Brosse, S. (2017) Seventy-five years of biodiversity decline of fish assemblages in Chinese isolated plateau lakes: widespread introductions and extirpations of narrow endemics lead to regional loss of dissimilarity. Diversity and Distributions, 23, 171-184.

Donazar, J.A., Gangoso, L., Forero, M.G. & Juste, J. (2005) Presence, richness and extinction of birds of prey in the Mediterranean and Macaronesian islands. Journal of Biogeography, 32, 1701-1713.

Dorst, J., Vuilleumier, F., Vuilleumier, F. & Monasterio, M. (1986) Convergences in bird communities at high altitudes in the Tropics (especially the Andes and Africa) and at high temperate latitudes (Tibet).

Dos Anjos, L. & Bocon, R. (1999) Bird communities in natural forest patches in southern Brazil. Wilson Bulletin, 111, 397-414.

dos Santos, J.P., Freitas, A.V.L., Brown, K.S., Carreira, J.Y.O., Gueratto, P.E., Rosa, A.H.B., Lourenco, G.M., Accacio, G.M., Uehara-Prado, M., Iserhard, C.A., Richter, A., Gawlinski, K., Romanowski, H.P., Mega, N.O., Teixeira, M.O., Moser, A., Ribeiro, D.B., Araujo, P.F., Filgueiras, B.K.C., Melo, D.H.A., Leal, I.R., Beirao, M.D., Ribeiro, S.P., Cambui, E.C.B., Vasconcelos, R.N., Cardoso, M.Z., Paluch, M., Greve, R.R., Voltolini, J.C., Galetti, M., Regolin, A.L., Sobral-Souza, T. & Ribeiro, M.C. (2018) Atlantic butterflies: a data set of fruit-feeding butterfly communities from the Atlantic forests. Ecology, 99, 2875-2875.

dos Santos, K., Kinoshita, L.S. & dos Santos, F.A.M. (2007) Tree species composition and similarity in semideciduous forest fragments of southeastern Brazil. Biological Conservation, 135, 268-277.

Dowsett, R.J., Vuilleumier, F. & Monasterio, M. (1986) Origins of the high-altitude avifaunas of tropical Africa.

Dueser, R.D. & Brown, W.C. (1980) Ecological correlates of insular rodent diversity. Ecology, 61, 50-56.

Edenius, L. & Sjoberg, K. (1997) Distribution of birds in natural landscape mosaics of old-growth forests in northern Sweden: relations to habitat area and landscape context. Ecography, 20, 425-431.

Enderson, E.F., Quijada-Mascarenas, A., Turner, D.S., Rosen, P.C. & Bezy, R.L. (2009) The herpetofauna of Sonora, Mexico, with comparisons to adjoining states. Check List, 5, 632-672.

Essl, F. & Dirnbock, T. (2012) What determines Orthoptera species distribution and richness in temperate semi-natural dry grassland remnants? Biodiversity and Conservation, 21, 2525-2537.

Fattorini, S. (2002) Biogeography of the tenebrionid beetles (Coleoptera, Tenebrionidae) on the Aegean Islands (Greece). Journal of Biogeography, 29, 49-67.

Feeley, K. (2003) Analysis of avian communities in Lake Guri, Venezuela, using multiple assembly rule models. Oecologia, 137, 104-113.

Fernandez-Juricic, E. (2000) Bird community composition patterns in urban parks of Madrid: The role of age, size and isolation. Ecological Research, 15, 373-383.

Filgueiras, B.K.C., Iannuzzi, L. & Leal, I.R. (2011) Habitat fragmentation alters the structure of dung beetle communities in the Atlantic Forest. Biological Conservation, 144, 362-369.

Flaspohler, D.J., Giardina, C.P., Asner, G.P., Hart, P., Price, J., Lyons, C.K. & Castaneda, X. (2010) Long-term effects of fragmentation and fragment properties on bird species richness in Hawaiian forests. Biological Conservation, 143, 280-288.

Ford, H.A. (1987) Bird communities on habitat islands in England. Bird Study, 34, 205-218.

Friday, L.E. (1987) The diversity of macroinvertebrate and macrophytes communities in ponds. Freshwater Biology, 18, 87-104.

Galle, R. (2008) The effect of a naturally fragmented landscape on the spider assemblages. North-Western Journal of Zoology, 4, 61-71.

Galli, A.E., Leck, C.F. & Forman, R.T.T. (1976) Avian distribution patterns in forest islands of different sizes in central New Jersey. Auk, 93, 356-364.

Gaublomme, E., Hendrickx, F., Dhuyvetter, H. & Desender, K. (2008) The effects of forest patch size and matrix type on changes in carabid beetle assemblages in an urbanized landscape. Biological Conservation, 141, 2585-2596.

Gavish, Y., Ziv, Y. & Rosenzweig, M.L. (2012) Decoupling Fragmentation from Habitat Loss for Spiders in Patchy Agricultural Landscapes. Conservation Biology, 26, 150-159.

Gibson, L.A. & McKenzie, N.L. (2012) Occurrence of non-volant mammals on islands along the Kimberley coast of Western Australia. Records of the Western Australian Museum, 15-40.

Gibson, L.A., Cowan, M.A., Lyons, M.N., Palmer, R., Pearson, D.J. & Doughty, P. (2017) Island refuges: Conservation significance of the biodiversity patterns resulting from 'natural' fragmentation. Biological Conservation, 212, 349-356.

Gotelli, N.J. & Abele, L.G. (1982) Statistical distributions of West Indian land bird families. Journal of Biogeography, 9, 421-435.

Götmark, F., Åhlund, M. & Eriksson, M.O.G. (1986) Are indices reliable for assessing conservation value of natural areas? An avian case study. Biological Conservation, 38, 55-73.

Gouveia, S.F. & Faria, R.G. (2015) Effects of Habitat Size and Heterogeneity on Anuran Breeding Assemblages in the Brazilian Dry Forest. Journal of Herpetology, 49, 442-446.

Graham, J.H. (1993) Species diversity of fishes in naturally acidic lakes in New Jersey. Transactions of the American Fisheries Society, 122, 1043-1057.

Granado-Lorencio, C., Serna, A.H., Carvajal, J.D., Jimenez-Segura, L.F., Gulfo, A. & Alvarez, F. (2012) Regionally nested patterns of fish assemblages in floodplain lakes of the Magdalena river (Colombia). Ecology and Evolution, 2, 1296-1303.

Gunn, I.D.M., O'Hare, M., Carvalho, L., Roy, D.B., Rothery, P. & Darwell, A.M. (2010) Assessing the condition of lake habitats: a test of methods for surveying aquatic macrophyte communities. Hydrobiologia, 656, 87-97.

Haila, Y. (1983) Land birds on northern islands - a sampling metaphor for insular colonization. Oikos, 41, 334-351.

Haila, Y., Jarvinen, O. & Vaisanen, R.A. (1980) Habitat distribution and species associations of land bird populations on the Aland Island, SW Finland. Annales Zoologici Fennici, 17, 87-106.

Haila, Y., Jarvinen, O. & Kuusela, S. (1983) Colonization of islands by land birds - prevalence functions in a Finnish archipelago. Journal of Biogeography, 10, 499-531.

Haila, Y., Hanski, I.K. & Raivio, S. (1987) Breeding bird distribution in fragmented coniferous taiga in southern Finland. Ornis Fennica, 64, 90-106.

Haila, Y., Hanski, I.K. & Raivio, S. (1993) Turnover of breeding birds in small forest fragments - the sampling colonization hypothesis corroborated. Ecology, 74, 714-725.

Harris, M.P. (1973) Galapogos avifauna. Condor, 75, 265-278.

Hattori, T. & Ishida, H. (2000) Relationship between species diversity, species composition and forest area of fragmented lucidophyllous forests in central Miyazaki Prefecture. Japanese Journal of Ecology (Otsu), 50, 221-234.

Hausdorf, B. & Hennig, C. (2005) The influence of recent geography, palaeogeography and climate on the composition of the fauna of the central Aegean Islands. Biological Journal of the Linnean Society, 84, 785-795.

Heaney, L.R. (1986) Biogeography of mammals in SE Asia - estimates of rates of colonization, extinction and speciation. Biological Journal of the Linnean Society, 28, 127-165.

Hirao, T., Kubota, Y. & Murakami, M. (2015) Geographical patterns of butterfly species diversity in the subtropical Ryukyu Islands: the importance of a unidirectional filter between two source islands. Journal of Biogeography, 42, 1418-1430.

Hitchin, G.G., Wile, I., Miller, G.E. & Yan, N.D. (1984) Macrophyte data from 46 southern Ontario soft-water lakes of varying pH. In. Report DR 84/2, Ont. Min. Environ. Data, Ontario, Canada.

Holbech, L.H. (2005) The implications of selective logging and forest fragmentation for the conservation of avian diversity in evergreen forests of south-west Ghana. Bird Conservation International, 15, 27-52.

Howe, R.W. & Jones, G. (1977) Avian utilization of small woodlots in Dane County, Wisconsin. Passenger Pigeon, 39, 313-319,illust.

Hu, G., Wu, J.G., Feeley, K.J., Xu, G.F. & Yu, M.J. (2012) The Effects of Landscape Variables on the Species-Area Relationship during Late-Stage Habitat Fragmentation. Plos One, 7

Ishida, H., Hattori, T., Takeda, Y. & Kodate, S. (1998) Relationship between species richness or species composition and area of fragmented lucidophyllous forests in southeastern Hyogo Prefecture. Japanese Journal of Ecology (Tokyo), 48, 1-16.

Jackson, S.T. & Charles, D.F. (1988) Aquatic macrophytes in Adirondack (New York) USA: lakes patterns of species composition in relation to environment. Canadian Journal of Botany, 66, 1449-1460.

Johnson, N.K. (1975) Controls of number of bird species on montane islands in Great-Basin. Evolution, 29, 545-567.

Kelt, D.A. (2000) Small mammal communities in rainforest fragments in Central Southern Chile. Biological Conservation, 92, 345-358.

King, J.L., Simovich, M.A. & Brusca, R.C. (1996) Species richness, endemism and ecology of crustacean assemblages in northern California vernal pools. Hydrobiologia, 328, 85-116.

Kitchener, D.J., Chapman, A., Muir, B.G. & Palmer, M. (1980a) The conservation value for mammals of reserves in the western Australian wheatbelt. Biological Conservation, 18, 179-207.

Kitchener, D.J., Chapman, A., Dell, J. & Muir, B.G. (1980b) Lizard assemblage and reserve size and structure in the western Australian wheatbelt - some implications for conservation. Biological Conservation, 17, 25-62.

Kratter, A.W. (1992) Montane avian biogeography in southern California and Baja California. Journal of Biogeography, 19, 269-283.

Krystufek, B. & Kletecki, E. (2007) Biogeography of small terrestrial vertebrates on the Adriatic landbridge islands. Folia Zoologica, 56, 225-234.

Lion, M.B., Garda, A.A., Santana, D.J. & Fonseca, C.R. (2016) The Conservation Value of Small Fragments For Atlantic Forest Reptiles. Biotropica, 48, 265-275.

Liu, J.L., Matthews, T.J., Zhong, L., Liu, J.J., Wu, D.H. & Yu, M.J. (2019) Environmental filtering underpins the island species-area relationship in a subtropical anthropogenic archipelago. Journal of Ecology,

Lomolino, M.V. (1986) Mammalian community structure on islands - the importance of immigration, extinction and interactive effects. Biological Journal of the Linnean Society, 28, 1-21.

Lomolino, M.V. & Davis, R. (1997) Biogeographic scale and biodiversity of mountain forest mammals of western North America. Global Ecology and Biogeography Letters, 6, 57-76.

Lomolino, M.V. & Perault, D.R. (2001) Island biogeography and landscape ecology of mammals inhabiting fragmented, temperate rain forests. Global Ecology and Biogeography, 10, 113-132.

Lomolino, M.V., Brown, J.H. & Davis, R. (1989) Island biogeography of montane forest mammals in the American southwest. Ecology, 70, 180-194.

Lumaret, R., Guillerm, J.L., Maillet, J. & Verlaque, R. (1997) Plant species diversity and polyploidy in islands of natural vegetation isolated in extensive cultivated lands. Biodiversity and Conservation, 6, 591-613.

MacDonald, Z.G., Anderson, I.D., Acorn, J.H. & Nielsen, S.E. (2018a) Decoupling habitat fragmentation from habitat loss: butterfly species mobility obscures fragmentation effects in a naturally fragmented landscape of lake islands. Oecologia, 186, 11-27.

MacDonald, Z.G., Anderson, I.D., Acorn, J.H. & Nielsen, S.E. (2018b) The theory of island biogeography, the sample-area effect, and the habitat diversity hypothesis: complementarity in a naturally fragmented landscape of lake islands. Journal of Biogeography, 45, 2730-2743.

Macias-Hernandez, N., Lopez, S.D., Roca-Cusachs, M., Oromi, P. & Arnedo, M.A. (2016) A geographical distribution database of the genus Dysdera in the Canary Islands (Araneae, Dysderidae). Zookeys, 11-23.

Marini, M.A. (2001) Effects of forest fragmentation on birds of the cerrado region, Brazil. Bird Conservation International, 11, 13-25.

Markwell, K.A. & Fellows, C.S. (2008) Habitat and biodiversity of on-farm water storages: A case study in Southeast Queensland, Australia. Environmental Management, 41, 234-249.

Marshall, A.R., Jorgensbye, H.I.O., Rovero, F., Platts, P.J., White, P.C.L. & Lovett, J.C. (2010) The Species Area Relationship and Confounding Variables in a Threatened Monkey Community. American Journal of Primatology, 72, 325-336.

Marsland, K. & Nicol, J. (2007) 2006 River Murray Wetlands Baseline Survey. Vegetation Component, Publication Number RD04/0245-4. South Australian Research and Development Institute (Aquatic Sciences) SARDI, Adelaide.

Martin, J.L. (1983) Impoverishment of island bird communities in a Finnish archipelago. Ornis Scandinavica, 14, 66-77.

Martin, J.L., Gaston, A.J. & Hitier, S. (1995) The effect of island size and isolation on old-growth forest habitat and bird diversity in Gwaii Haanas (Queen Charlotte Islands, Canada). Oikos, 72, 115-131.

Matthiae, P.E. & Stearns, F. (1981) Mammals in forest islands in southeastern Wisconsin. Ecological Studies, 41, 55-66.

McCollin, D. (1993) Avian distribution patterns in a fragmented wooded landscape (North Humberside, UK) - the role of between-patch and within-patch structure. Global Ecology and Biogeography Letters, 3, 48-62.

McKenzie, N.L. & Bullen, R.D. (2012) An acoustic survey of zoophagic bats on islands in the Kimberley, Western Australia, including data on the echolocation ecology, organisation and habitat relationships of regional communities. Records of the Western Australian Museum, 67-108.

Meynard, C.N. & Quinn, J.F. (2008) Bird metacommunities in temperate South American forest: Vegetation structure, area, and climate effects. Ecology, 89, 981-990.

Mitsuo, Y., Tsunoda, H., Ohira, M., Doi, M. & Senga, Y. (2011) Nested subset patterns of species composition in a pond-dwelling fish fauna. Ecological Research, 26, 311-316.

Miyashita, T., Shinkai, A. & Chida, T. (1998) The effects of forest fragmentation on web spider communities in urban areas. Biological Conservation, 86, 357-364.

Mohd-Azlan, J. & Lawes, M.J. (2011) The effect of the surrounding landscape matrix on mangrove bird community assembly in north Australia. Biological Conservation, 144, 2134-2141.

Newmark, W.D. (1991) Tropical forest fragmentation and the local extinction of understory birds in the eastern Usambara Mountains, Tanzania. Conservation Biology, 5, 67-78.

Nores, M. (1995) Insular biogeography of birds on mountain-tops in north-western Argentina. Journal of Biogeography, 22, 61-70.

Nufio, C.R., McClenahan, J.L. & Thurston, E.G. (2009) Determining the effects of habitat fragment area on grasshopper species density and richness: a comparison of proportional and uniform sampling methods. Insect Conservation and Diversity, 2, 295-304.

Nufio, C.R., McClenahan, J.L. & Bowers, M.D. (2011) Grasshopper response to reductions in habitat area as mediated by subfamily classification and life history traits. Journal of Insect Conservation, 15, 409-419.

Nyeko, P. (2009) Dung Beetle Assemblages and Seasonality in Primary Forest and Forest Fragments on Agricultural Landscapes in Budongo, Uganda. Biotropica, 41, 476-484.

Panitsa, M. & Tzanoudakis, D. (2010) Floristic diversity on small islands and islets: Leros islets' group (East Aegean area, Greece). Phytologia Balcanica, 16, 271-284.

Patterson, B.D. (1984) Mammalian extinction and biogeography in the southern Rocky Mountains.

Pavuk, D.M. & Wadsworth, A.M. (2012) Longhorned beetle (Coleoptera: Cerambycidae) diversity in a fragmented temperate forest landscape. F1000Research, 1, 25.

Peltzer, P.M., Lajmanovich, R.C. & Beltzer, A. (2003) The effect of habitat fragmentation on amphibian species richness in the floodplain of the Middle Parana River, Argentina. Herpetological Journal, 13, 95-98.

Pineda, E. & Halffter, G. (2004) Species diversity and habitat fragmentation: frogs in a tropical montane landscape in Mexico. Biological Conservation, 117, 499-508.

Power, D.M. (1972) Numbers of bird species on California islands. Evolution, 26, 451-&.

Presley, S.J. & Willig, M.R. (2008) Composition and structure of Caribbean bat (Chiroptera) assemblages: effects of inter-island distance, area, elevation and hurricane-induced disturbance. Global Ecology and Biogeography, 17, 747-757.

Presley, S.J. & Willig, M.R. (2010) Bat metacommunity structure on Caribbean islands and the role of endemics. Global Ecology and Biogeography, 19, 185-199.

Ramanamanjato, J.B., McIntyre, P.B. & Nussbaum, R.A. (2002) Reptile, amphibian, and lemur diversity of the Malahelo Forest, a biogeographical transition zone in southeastern Madagascar. Biodiversity and Conservation, 11, 1791-1807.

Ribas, C.R., Sobrinho, T.G., Schoereder, J.H., Sperber, C.F., Lopes-Andrade, C. & Soares, S.M. (2005) How large is large enough for insects? Forest fragmentation effects at three spatial scales. Acta Oecologica-International Journal of Ecology, 27, 31-41.

Rizali, A., Lohman, D.J., Buchori, D., Prasetyo, L.B., Triwidodo, H., Bos, M.M., Yamane, S. & Schulze, C.H. (2010) Ant communities on small tropical islands: effects of island size and isolation are obscured by habitat disturbance and 'tramp' ant species. Journal of Biogeography, 37, 229-236.

Rosenblatt, D.L., Heske, E.J., Nelson, S.L., Barber, D.H., Miller, M.A. & MacAllister, B. (1999) Forest fragments in east-central Illinois: Islands or habitat patches for mammals? American Midland Naturalist, 141, 115-123.

Rusterholz, K.A. & Howe, R.W. (1979) Species-area relations of birds on small islands in a Minnesota lake. Evolution, 33, 468-477.

Schlaepfer, M.A. & Gavin, T.A. (2001) Edge effects on lizards and frogs in tropical forest fragments. Conservation Biology, 15, 1079-1090.

Sebastian-Gonzalez, E., Molina, J.A. & Paracuellos, M. (2012) Distribution patterns of a marsh vegetation metacommunity in relation to habitat configuration. Aquatic Biology, 16, 277-285.

Shreeve, T.G. & Mason, C.F. (1980) The number of butterfly species in woodlands. Oecologia, 45, 414-418.

Silva, M. (2001) Abundance, diversity, and community structure of small mammals in forest fragments in Prince Edward Island National Park, Canada. Canadian Journal of Zoology-Revue Canadienne De Zoologie, 79, 2063-2071.

Silva, M.P.P. & Porto, K.C. (2009) Effect of fragmentation on the community structure of epixylic bryophytes in Atlantic Forest remnants in the Northeast of Brazil. Biodiversity and Conservation, 18, 317-337.

Simberloff, D. (1976) Experimental zoogeography of islands - effects of island size. Ecology, 57, 629-648.

Simberloff, D. & Martin, J.L. (1991) Nestedness of insular avifaunas - simple summary statistics masking complex species patterns. Ornis Fennica, 68, 178-192.

Smith, B.B. & Fleer, D. (2007) Final report on the ‘Fish’ and ‘Water Quality’ components of the 2006 River Murray Wetlands Baseline Survey RD 04/0245-3. In, p. 44, Adelaide, South Australia.

Smith, G.T., Arnold, G.W., Sarre, S., AbenspergTraun, M. & Steven, D.E. (1996) The effects of habitat fragmentation and livestock-grazing on animal communities in remnants of gimlet Eucalyptus salubris woodland in the Western Australian wheatbelt .2. Lizards. Journal of Applied Ecology, 33, 1302-1310.

Soga, M., Ishiyama, N., Sueyoshi, M., Yamaura, Y., Hayashida, K., Koizumi, I. & Negishi, J.N. (2014) Interaction between patch area and shape: lakes with different formation processes have contrasting area and shape effects on macrophyte diversity. Landscape and Ecological Engineering, 10, 55-64.

Soule, M. & Sloan, A.J. (1966) Biogeography and distribution of the reptiles and amphibians on islands in the Gulf of California, Mexico. Trans San Diego Soc Natur Hist, 14, 137-156.

Soule, M.E., Bolger, D.T., Alberts, A.C., Wright, J., Sorice, M. & Hill, S. (1988) Reconstructed dynamics of rapid extinctions of chaparral-requiring birds in urban habitat islands. Conservation Biology, 2, 75-92.

Spengler, A., Hartmann, P., Buchori, D. & Schulze, C.H. (2011) How island size and isolation affect bee and wasp ensembles on small tropical islands: a case study from Kepulauan Seribu, Indonesia. Journal of Biogeography, 38, 247-258.

SSWDB (2017) Scottish Standing Waters Standing Waters Database, Scottish Natural Heritage, accessed 13 September 2017, URL: http://gateway.snh.gov.uk/pls/apex_cagdb2/f?p=111:1000:::NO:

Stamou, G., Polyzou, C., Karagianni, A. & Michaloudi, E. (2017) Taxonomic distinctness indices for discriminating patterns in freshwater rotifer assemblages. Hydrobiologia, 796, 319-331.

Suarez, A.V., Bolger, D.T. & Case, T.J. (1998) Effects of fragmentation and invasion on native ant communities in coastal southern California. Ecology, 79, 2041-2056.

Surendran, H. & Vasudevan, K. (2015) The devil is in the detail: estimating species richness, density, and relative abundance of tropical island herpetofauna. Bmc Ecology, 15

Tennent, W.J. & Russell, P.J.C. (2014) Butterflies of the Cape Verde Islands (Insecta, Lepidoptera). Zoologia Caboverdiana, 5, 64-104.

Thibault, J.C., Martin, J.L. & Guyot, I. (1990) The breeding land birds of the small islands in the Strait of Bonifacio population analysis. Alauda, 58, 173-185.

Thornhill, I., Batty, L., Death, R.G., Friberg, N.R. & Ledger, M.E. (2017) Local and landscape scale determinants of macroinvertebrate assemblages and their conservation value in ponds across an urban land-use gradient. Biodiversity and Conservation, 26, 1065-1086.

Tonn, W.M. & Magnuson, J.J. (1982) Patterns in the species composition and richness of fish assemblages in northern Wisconsin lakes. Ecology, 63, 1149-1166.

Urban, M.C. (2004) Disturbance heterogeneity determines freshwater metacommunity structure. Ecology, 85, 2971-2978.

Usher, M.B. & Keiller, S.W.J. (1998) The macrolepidoptera of farm woodlands: determinants of diversity and community structure. Biodiversity and Conservation, 7, 725-748.

Vallan, D. (2000) Influence of forest fragmentation on amphibian diversity in the nature reserve of Ambohitantely, highland Madagascar. Biological Conservation, 96, 31-43.

Waanders, P. (2007) River Murray wetland bird surveys. In: Draft report on the Bird survey component of the 2006 SA River Murray Wetlands Baseline Survey

Wang, Y.P., Ding, P., Chen, S.H. & Zheng, G.M. (2013) Nestedness of bird assemblages on urban woodlots: Implications for conservation. Landscape and Urban Planning, 111, 59-67.

Wang, Y.P., Bao, Y.X., Yu, M.J., Xu, G.F. & Ding, P. (2010) Nestedness for different reasons: the distributions of birds, lizards and small mammals on islands of an inundated lake. Diversity and Distributions, 16, 862-873.

Watson, D.M. (2003) Long-term consequences of habitat fragmentation - highland birds in Oaxaca, Mexico. Biological Conservation, 111, 283-303.

Weaver, M. & Kellman, M. (1981) The effects of forest fragmentation on woodlot tree biotas in southern Ontario. Journal of Biogeography, 8, 199-210.

Weller, B. & Ganzhorn, J.U. (2004) Carabid beetle community composition, body size, and fluctuating asymmetry along an urban-rural gradient. Basic and Applied Ecology, 5, 193-201.

Wilson, M.F., De Santo, T.L., Sabag, C. & Armesto, J.J. (1994) Avian communities of fragmented south-temperate rainforests in Chile. Conservation Biology, 8, 508-520.

Wright, S.J. (1985) How isolation affects rates of turnover of species on islands. Oikos, 44, 331-340.

Xu, A.C., Han, X.F., Zhang, X.M., Millien, V. & Wang, Y.P. (2017) Nestedness of butterfly assemblages in the Zhoushan Archipelago, China: area effects, life-history traits and conservation implications. Biodiversity and Conservation, 26, 1375-1392.

Yu, J., Shen, L., Li, D.D. & Guo, S.L. (2019) Determinants of bryophyte species richness on the Zhoushan Archipelago, China. Basic and Applied Ecology, 37, 38-50.

Zhang, X., Han, X., Liu, L. & Xu, A. (2016) Influencing factors of the nested distribution of butterfly assemblages in the Zhoushan Archipelago, China. Biodiversity Science, 24, 321-331.

# References for supporting information

Aldasoro JJ, Cabezas F, Aedo C (2004) Diversity and distribution of ferns in sub-Saharan Africa, Madagascar and some islands of the South Atlantic. Journal of Biogeography 31:1579-1604. doi: 10.1111/j.1365-2699.2004.01106.x

Chase JM et al. (2019) A framework for disentangling ecological mechanisms underlying the island species-area relationship. Frontiers of Biogeography 11:e40844. doi: 10.21425/F5FBG40844

Fahrig L (2017) Ecological Responses to Habitat Fragmentation Per Se. In: Futuyma DJ (ed) Annual Review of Ecology, Evolution, and Systematics, Vol 48, vol 48. Annual Reviews, Palo Alto, pp 1-23

Fletcher RJ et al. (2018) Is habitat fragmentation good for biodiversity? Biological Conservation 226:9-15. doi: 10.1016/j.biocon.2018.07.022

Gavish Y, Ziv Y, Rosenzweig ML (2012) Decoupling Fragmentation from Habitat Loss for Spiders in Patchy Agricultural Landscapes. Conservation Biology 26:150-159. doi: 10.1111/j.1523-1739.2011.01799.x

Mac Nally R, Lake PS (1999) On the generation of diversity in archipelagos: a re-evaluation of the Quinn-Harrison 'saturation index'. Journal of Biogeography 26:285-295. doi: 10.1046/j.1365-2699.1999.00268.x

Quinn JF, Harrison SP (1988) Effects of habitat fragmentation and isolation on species richness - evidence from biogeographic patterns. Oecologia 75:132-140. doi: 10.1007/bf00378826

Ramsey FL (1989) Comments on a saturation index. Oecologia 81:569-570. doi: 10.1007/bf00378971

Whittaker RH (1960) Vegetation of the Siskiyou Mountains, Oregon and California. Ecological Monographs 30:280-338
