# Supplementary material for: Species accumulation in small–large vs large–small order: more species but not all species?
Source: Oecologia. 2022 Sep 17;200(1-2):273–84. doi: 10.1007/s00442-022-05261-1 (PMC9547801; doi:10.1007/s00442-022-05261-1)
Supplement: Supplementary file 4 — Supplementary file4 (DOCX 26 KB) [file 442_2022_5261_MOESM4_ESM.docx]

Supporting information for

**Species accumulation in small-large vs large-small order: more species but not all species?**

DC Deane

# Online resource 6: Appendix S6 - Subdivision criteria for mutually exclusive conditions

As there are two curves, each with four possible outcomes, there are 2^4^ = 16 possible mutually exclusive logical combinations of observed and expected values (Table S6.1). Small-patch dominance (where more species than implied by passive sampling were found when combining small-large patches) is inferred for conditions 3, 5, 7, 8 and 15 with a total number of datasets of 37 (18%). Large-patch dominance (where more species than are implied by passive sampling is observed when combining large-small patches) is inferred for conditions 2, 9, 10, 12 and 14, with a total number of datasets of 66 (33%). The number of datasets where small-large and large-small accumulation both fall within expected range corresponds to condition 16 with a total of 81 datasets (40%). Under these criteria, 9% of datasets remain indeterminate (conditions 1, 4, 6, 11 and 13) and these were omitted from post hoc analyses.

**Table S6.1** Assignment of 16 possible mutually exclusive states of the null model simulations to hypotheses and frequency of observation of each state in simulations. Each cell gives the logical condition (>0 means more than one point falls outside the corresponding interval for small-large (S-L) or large-small (L-S) order of patch combination. H_0_ = null hypothesis of passive sampling; H_AL_ = alternative hypothesis, indicating disproportionate importance of large patches for species accumulation; H_AS_ = alternative hypothesis indicating disproportionate importance of small patches for species accumulation; Indet = indeterminant inference for patch-size dependence on species accumulation.

| Condition | S-L  upper CI | S-L  lower CI | L-S  upper CI | L-S  lower CI | Count | Hypothesis |
| --- | --- | --- | --- | --- | --- | --- |
| 1 | >0 | >0 | >0 | >0 | 1 | Indet |
| 2 | >0 | >0 | >0 | 0 | 0 | H_AL_ |
| 3 | >0 | >0 | 0 | >0 | 3 | H_AS_ |
| 4 | >0 | >0 | 0 | 0 | 2 | Indet |
| 5 | >0 | 0 | >0 | >0 | 1 | H_AS_ |
| 6 | >0 | 0 | >0 | 0 | 0 | Indet |
| 7 | >0 | 0 | 0 | >0 | 8 | H_AS_ |
| 8 | >0 | 0 | 0 | 0 | 9 | H_AS_ |
| 9 | 0 | >0 | >0 | >0 | 6 | H_AL_ |
| 10 | 0 | >0 | >0 | 0 | 23 | H_AL_ |
| 11 | 0 | >0 | 0 | >0 | 15 | Indet |
| 12 | 0 | >0 | 0 | 0 | 33 | H_AL_ |
| 13 | 0 | 0 | >0 | >0 | 0 | Indet |
| 14 | 0 | 0 | >0 | 0 | 4 | H_AL_ |
| 15 | 0 | 0 | 0 | >0 | 16 | H_AS_ |
| 16 | 0 | 0 | 0 | 0 | 81 | H_0_ |

# Online resource 7: Appendix S7 - Supporting results

Table S7.1 Proportion of datasets assigned to different diversity outcomes using overlap criteria from QH curves (SLOSS analysis) and hypothesis tests for passive sampling null model (Simulations). Outcome STL indicates small-to-large accumulation was greater than expected (corresponding to alternative hypothesis H_AS_), ND indicates no difference (corresponding to failure to reject H_0_); LTS indicates large-to-small accumulation was greater than expected (corresponding to H_AL_). See Methods and Appendix S6 for explanation of how categories were assigned. ‘Patch’ refers to different metacommunity types, with level abbreviations: Ar = archipelago, i.e., true island system; Fr = fragments of formerly contiguous biomes; Ha = habitat island (e.g., wetland). ‘Taxa’ refers to broad taxonomic groups, with level abbreviations: BRD = birds, INV = invertebrates, PLT = plants and fungi, VER = non-avian vertebrates.

| Factor | Level | SLOSS analysis | | | Null models | | |
| --- | --- | --- | --- | --- | --- | --- | --- |
|  |  | STL | ND | LTS | H_AS_ | H_0_ | H_AL_ |
| Patch | Ar | 0.23 | 0.09 | 0.02 | 0.06 | 0.11 | 0.16 |
|  | Fr | 0.26 | 0.10 | 0.01 | 0.05 | 0.20 | 0.08 |
|  | Ha | 0.18 | 0.06 | 0.04 | 0.07 | 0.09 | 0.08 |
| Taxa | BRD | 0.17 | 0.08 | 0.04 | 0.07 | 0.09 | 0.09 |
|  | INV | 0.26 | 0.06 | 0.01 | 0.12 | 0.10 | 0.08 |
|  | PLT | 0.13 | 0.02 | 0.00 | 0.02 | 0.04 | 0.07 |
|  | VER | 0.12 | 0.10 | 0.02 | 0.01 | 0.17 | 0.08 |
| Total: | | 0.67 | 0.26 | 0.07 | 0.18 | 0.40 | 0.33 |

# References for supporting information

BÁLDI, A. & KISBENEDEK, T. 1999. Orthopterans in small steppe patches: an investigation for the best-fit model of the species-area curve and evidences for their non-random distribution in the patches. *Acta Oecologica-International Journal of Ecology,* 20**,** 125-132.

CHASE, J. M., GOORIAH, L., MAY, F., RYBERG, W. A., SCHULER, M. S., CRAVEN, D. & KNIGHT, T. M. 2019. A framework for disentangling ecological mechanisms underlying the island species-area relationship. *Frontiers of Biogeography,* 11.

FAHRIG, L. 2017. Ecological Responses to Habitat Fragmentation Per Se. *In:* FUTUYMA, D. J. (ed.) *Annual Review of Ecology, Evolution, and Systematics, Vol 48.* Palo Alto: Annual Reviews.

FLETCHER, R. J., DIDHAM, R. K., BANKS-LEITE, C., BARLOW, J., EWERS, R. M., ROSINDELL, J., HOLT, R. D., GONZALEZ, A., PARDINI, R., DAMSCHEN, E. I., MELO, F. P. L., RIES, L., PREVEDELLO, J. A., TSCHARNTKE, T., LAURANCE, W. F., LOVEJOY, T. & HADDAD, N. M. 2018. Is habitat fragmentation good for biodiversity? *Biological Conservation,* 226**,** 9-15.

GAVISH, Y., ZIV, Y. & ROSENZWEIG, M. L. 2012. Decoupling Fragmentation from Habitat Loss for Spiders in Patchy Agricultural Landscapes. *Conservation Biology,* 26**,** 150-159.

MAC NALLY, R. & LAKE, P. S. 1999. On the generation of diversity in archipelagos: a re-evaluation of the Quinn-Harrison 'saturation index'. *Journal of Biogeography,* 26**,** 285-295.

QUINN, J. F. & HARRISON, S. P. 1988. Effects of habitat fragmentation and isolation on species richness - evidence from biogeographic patterns. *Oecologia,* 75**,** 132-140.

RAMSEY, F. L. 1989. Comments on a saturation index. *Oecologia,* 81**,** 569-570.

WHITTAKER, R. H. 1960. Vegetation of the Siskiyou Mountains, Oregon and California. *Ecological Monographs,* 30**,** 280-338.

BÁLDI, A. & KISBENEDEK, T. 1999. Orthopterans in small steppe patches: an investigation for the best-fit model of the species-area curve and evidences for their non-random distribution in the patches. *Acta Oecologica-International Journal of Ecology,* 20**,** 125-132.

FAHRIG, L. 2017. Ecological Responses to Habitat Fragmentation Per Se. *In:* FUTUYMA, D. J. (ed.) *Annual Review of Ecology, Evolution, and Systematics, Vol 48.* Palo Alto: Annual Reviews.

FLETCHER, R. J., DIDHAM, R. K., BANKS-LEITE, C., BARLOW, J., EWERS, R. M., ROSINDELL, J., HOLT, R. D., GONZALEZ, A., PARDINI, R., DAMSCHEN, E. I., MELO, F. P. L., RIES, L., PREVEDELLO, J. A., TSCHARNTKE, T., LAURANCE, W. F., LOVEJOY, T. & HADDAD, N. M. 2018. Is habitat fragmentation good for biodiversity? *Biological Conservation,* 226**,** 9-15.

GAVISH, Y., ZIV, Y. & ROSENZWEIG, M. L. 2012. Decoupling Fragmentation from Habitat Loss for Spiders in Patchy Agricultural Landscapes. *Conservation Biology,* 26**,** 150-159.

MAC NALLY, R. & LAKE, P. S. 1999. On the generation of diversity in archipelagos: a re-evaluation of the Quinn-Harrison 'saturation index'. *Journal of Biogeography,* 26**,** 285-295.

QUINN, J. F. & HARRISON, S. P. 1988. Effects of habitat fragmentation and isolation on species richness - evidence from biogeographic patterns. *Oecologia,* 75**,** 132-140.

RAMSEY, F. L. 1989. Comments on a saturation index. *Oecologia,* 81**,** 569-570.

WHITTAKER, R. H. 1960. Vegetation of the Siskiyou Mountains, Oregon and California. *Ecological Monographs,* 30**,** 280-338.
